# Knowledge, Attitudes, and Behaviors of Italian Home Care Nurses: Factors Associated with Medication Error Prevention in a Nationwide Cross-Sectional Survey

**DOI:** 10.3390/nursrep16030098

**Published:** 2026-03-14

**Authors:** Sara Dionisi, Emanuele Di Simone, Aurora De Leo, Gloria Liquori, Nicolò Panattoni, Leandro Amato, Alessandra Improta, Sofia Taborri, Giovanni Battista Orsi, Noemi Giannetta, Marco Di Muzio

**Affiliations:** 1 Nursing, Technical, Rehabilitation Department, DaTeR Azienda Unità Sanitaria Locale di Bologna, 40124 Bologna, Italy; sara.dionisi2@unibo.it; 2 Department of Medical and Movement Sciences and Wellness, Parthenope University of Naples, 80133 Naples, Italy; emanuele.disimone@uniparthenope.it; 3 Nursing Research Unit IFO, IRCCS Regina Elena National Cancer Institute, 00144 Rome, Italy; aurora.deleo@ifo.it (A.D.L.); nicolo.panattoni@ifo.it (N.P.); 4 Department of Emergency and Acceptance (DEA), Local Health Authority ASL Roma 1, 00135 Rome, Italy; gloria.liquori@uniroma1.it; 5 Department of Biomedicine and Prevention, University of Rome Tor Vergata, 00133 Rome, Italy; leandro.amato@uniroma1.it (L.A.); als.improta@aslsalerno.it (A.I.); sofia.taborri@uniroma1.it (S.T.); 6 Department of Public Health and Infectious Diseases, Sapienza University of Rome, 00185 Rome, Italy; giovanni.orsi@uniroma1.it; 7 Departmental Faculty of Medicine, Saint Camillus International University of Health Sciences, UniCamillus, 00131 Rome, Italy; 8 Department of Wellbeing, Health and Environmental Sustainability, Sapienza University of Rome, 00185 Rome, Italy; marco.dimuzio@uniroma1.it

**Keywords:** medication errors, home care, nursing, knowledge, attitudes, behaviors, logistic regression, patient safety

## Abstract

**Objectives:** This study aims to identify factors associated with medication errors among home care nurses in Italy, focusing on the relationships between knowledge, attitudes, and behaviors, and assessing how sociodemographic and professional characteristics influence these dimensions. **Methods**: A nationwide cross-sectional survey was conducted using the Italian validated version of the Knowledge, Attitudes, and Behaviors in Medication Error in the Home Care setting questionnaire, previously developed and validated for home care settings. Multivariate logistic regression analyses were performed to explore associations among knowledge, attitudes, behaviors, and selected sociodemographic variables because the survey was disseminated through open online channels, and the response rate could not be calculated. **Results:** A total of 320 nurses participated. Younger age and holding a non-university degree were significantly associated with higher knowledge levels. Internet access at the workplace emerged as the only significant factor associated with medication error prevention for both positive attitudes [OR = 0.412, 95% CI: 0.197–0.861; *p* = 0.018] and correct behaviors [OR = 0.456, 95% CI: 0.216–0.962; *p* = 0.039]. Furthermore, attitudes positively predicted knowledge [OR = 2.226, 95% CI: 1.291–3.962, *p* = 0.004], and both knowledge and attitudes significantly influenced behaviors. **Conclusions:** The study highlights the interdependence of knowledge, attitudes, and behaviors in preventing medication errors in home care. While internet access and formal education are associated with differences in knowledge, attitudes, and behaviors, the relationships observed warrant further investigation. These findings underscore the potential value of targeted educational strategies and resource availability to support nurses in promoting safe practices.

## 1. Introduction

Medication errors are among the leading causes of adverse events in healthcare, with a significant impact on patient safety, particularly in the home care setting [1,2]. As healthcare systems evolve towards increasingly community-based models of care, the safe management of medication therapy at home has become a crucial priority [3]. In this context, nurses play a central role not only in administering medications but also in educating patients about their treatments, although they are frequently exposed to multiple risk factors that may contribute to the occurrence of medication errors [4]. Although some of the factors contributing to errors are shared across both hospital and home care settings, the way these are addressed differs significantly. Several studies indicate that specific characteristics of the home care population—such as advanced age, chronic conditions, dependence on others, and polypharmacy—make care and medication management more complex [5,6,7].

The complexity of home care is also reflected in the organizational and communicative aspects of therapeutic management. Unlike the hospital setting, where multidisciplinary teamwork is continuous and structured, home care relies heavily on direct relationships with patients, family members, and caregivers [8]. Limited and non-continuous access to key professionals, such as general practitioners or pharmacists, may restrict nurses’ autonomy in managing medication-related issues [6]. Difficulties in clarifying prescriptions or managing dosage changes can compromise continuity of care, particularly during care transitions from hospital to home, which are widely recognized as critical phases for medication safety [6,9]. Medication reconciliation is considered a key strategy to mitigate such risks [10,11], although its effectiveness may be limited by poor interprofessional communication and the lack of shared tools. In this regard, pharmacist involvement and the use of digital technologies, such as electronic prescriptions, have been identified as strategies to enhance therapeutic accuracy and information sharing [12].

Additional barriers to medication safety include the lack of updated resources, limited availability of digital tools, and inadequate continuing education opportunities for home care nurses—conditions that may compromise their level of knowledge, influence their attitudes, and affect their behavior in safe medication management [12,13]. Moreover, the home environment introduces additional variables—such as environmental distractions and lack of clinical supervision—that are not typically found in institutional settings. Scientific literature suggests that both personal factors—such as professional experience, educational level, and digital literacy—and systemic factors—such as workload, access to information, and patient complexity—can influence the incidence of medication errors [14]. However, most existing studies focus on hospital settings, while data related to home care remain limited, particularly at the national level [15].

Within this framework, the Italian context presents specific organizational characteristics that are relevant to medication safety in home care. In Italy, home care services are primarily provided through the Integrated Home Care (ADI) model, a social and healthcare service included in the Essential Levels of Care (LEA) of the National Health Service. ADI provides multidisciplinary home care to individuals with chronic conditions, frailty, or functional limitations who require ongoing healthcare interventions that cannot be delivered through outpatient care. Access to the service is generally initiated by general practitioners and coordinated by local health authorities through an individualized care plan developed by a multidisciplinary team. The inclusion of home care within the LEA has led to the development of a National Information System for the Monitoring of Home Care, designed to collect patient-centered data on health and social care interventions delivered in home settings. However, while this system provides an overview of home care provision at the national level, it does not include standardized indicators specifically focused on medication safety or structured reporting systems for medication errors in home care.

Furthermore, the organization of home care services in Italy is characterized by a highly decentralized governance structure. Although national legislation defines the general framework for home care services, their organization and implementation are the responsibility of regional authorities and local health organizations. This results in substantial heterogeneity across regions in terms of care models, professional roles, and available resources, which may further influence medication management practices in home care.

Finally, the organization and delivery of home care services have been profoundly affected by the COVID-19 pandemic, which increased the demand for home-based care and intensified nurses’ workload and organizational complexity. In this context, the strategic role of community-based healthcare has been further reinforced by national health policies, including the National Recovery and Resilience Plan (PNRR), which explicitly promotes the strengthening of home care as a key setting for healthcare delivery.

In this context, and drawing on theoretical frameworks and previous studies that conceptualize medication safety as the result of the interaction between knowledge, attitudes, and behaviors, as well as empirical evidence showing that these domains are associated with safer practices and reporting behaviors, this study aims to examine factors associated with the prevention of medication errors among nurses working in Italian home care settings [13,16]. Specifically, it explores how sociodemographic and professional characteristics—such as age, education, and access to digital resources—are associated with nurses’ knowledge, attitudes, and behaviors regarding medication safety. Furthermore, the study examines the interrelationships among these three domains, in order to identify how they may support safer clinical practices in medication management within the home care setting. Based on the knowledge, attitudes and behaviours framework, we hypothesised that higher knowledge would be associated with more positive attitudes and that higher knowledge and more positive attitudes would be associated with safer behaviours related to medication error prevention. Associations between KAB (Knowledge, Attitudes, Behaviors) domains and selected sociodemographic and professional characteristics were explored as hypothesis-generating, given the observational design.

## 2. Materials and Methods

### 2.1. Design of the Study

This is a national cross-sectional study with an exploratory design. The study follows the STROBE (Strengthening the Reporting of Observational Studies in Epidemiology) reporting guidelines.

### 2.2. Participants and Study Setting

A non-probabilistic convenience sampling strategy was used to recruit participants. This strategy was adopted because no national registry reports the total number of nurses working in home care services in Italy. Therefore, it was not possible to define a sampling frame for probability-based sampling.

All nurses working in home care who, according to the care model adopted, are involved in the process of managing drug therapy were eligible to participate in the study. In addition, both nurses holding an academic nursing degree and nurses holding a legally recognised equivalent pre-university nursing qualification were eligible to participate in the study. Non-nursing healthcare professionals, those working in settings other than the patient’s home, and those with less than one month’s service were excluded from the study. Eligibility was assessed through self-reported screening questions administered immediately after the informed consent section. To reduce the risk of misclassification, the questionnaire included screening questions regarding professional role, workplace setting, and years of work experience. Responses not meeting the inclusion criteria were excluded during data cleaning.

The survey was distributed nationwide throughout Italy, and participants were recruited from different geographical macro-areas (northern, central, and southern Italy), supporting the national scope of the study.

Since no national registry reports the total number of nurses specifically working in home care services in Italy, it was not possible to estimate the target population size or perform an a priori sample size calculation. For this reason, the study was designed as an exploratory national survey aimed at collecting preliminary evidence on medication safety in home care. Therefore, the results should be interpreted as exploratory and hypothesis-generating rather than confirmatory.

### 2.3. Study Variables

The survey instrument used was the Knowledge, Attitudes, and Behaviors in Medication Error in the Home Care setting (KABMeHoQ) questionnaire, which has been validated in Italian for the home care setting [17,18]. The validation study showed satisfactory psychometric properties and good internal consistency for the overall scale (Cronbach’s alpha = 0.772) [19].

The online survey form included two sections. The first section collected participants’ professional and sociodemographic characteristics (e.g., age, gender, educational pathway, years of work experience, geographic area, workplace internet access, and training in medication management). The second section consists of 20 items that investigate the knowledge, attitudes, and behaviors of nurses regarding strategies for reducing therapeutic errors in home care.

The knowledge section includes seven items rated on a five-point Likert scale (0 = strongly disagree, 1 = disagree, 2 = neither agree nor disagree, 3 = agree, 4 = strongly agree). Item scores were summed to obtain a total knowledge score ranging from 0 to 28, with higher scores indicating greater knowledge of medication error risk factors and preventive strategies. The attitude section consists of seven items rated on a three-point Likert scale (0 = disagree, 1 = partially agree, 2 = strongly agree). Item scores were summed to obtain a total attitude score ranging from 0 to 14; higher scores reflect more positive attitudes toward safe medication practices. The behavior section includes six items rated on a five-point Likert scale (0 = never, 1 = rarely, 2 = sometimes, 3 = often, 4 = always). Item scores were summed to obtain a total behavior score ranging from 0 to 24; higher scores indicate greater adherence to behaviors aimed at preventing medication errors.

The use of different Likert scales across questionnaire domains reflects the structure of the original validated instrument and was retained to preserve consistency with the questionnaire’s psychometric validation. The survey instrument has been previously described and validated in earlier studies [17,18] and is available from the authors upon request.

### 2.4. Data Collection Methods

Data were collected between September 2020 and March 2021 through an online web-based survey. Participants were recruited through social media and other online channels. The questionnaire was administered using the Google Forms^®^ platform and advertised on nursing-related websites and social media platforms to reach nursing professionals working in the home care setting. Because the survey was disseminated through open online channels and social media, it was not possible to determine the number of nurses who were reached by the invitation; therefore, a response rate could not be calculated.

The survey was self-administered and completed anonymously. To reduce ineligible participation, the survey included screening questions on healthcare setting and work experience; respondents not meeting the inclusion criteria were excluded during data cleaning.

### 2.5. Statistical Analysis

The socio-demographic and professional characteristics of the participants were analysed using descriptive statistics. Prior to analysis, the dataset was checked for completeness. No missing data were observed for the variables included in the regression analyses. Continuous variables were described using means and standard deviations or medians and interquartile ranges, depending on the distribution of the data. The distribution of variables was explored using the Shapiro–Wilk and Kolmogorov–Smirnov tests. For variables not following a normal distribution, non-parametric tests were used (Mann–Whitney U test for two-group comparisons and Kruskal–Wallis test for comparisons across more than two groups). When the Kruskal–Wallis test was statistically significant, post hoc pairwise comparisons were performed.

In accordance with the analysis model used by the author of the instrument and in previous observational studies adopting a similar approach [14,20], logistic regression analyses were conducted following recommendations for modelling in observational studies proposed by Hosmer and Lemeshow [21].

For these exploratory regression analyses, three dichotomous outcome variables were created: “adequate knowledge”, “positive attitudes” and “correct behaviours”. Participants who reported a medium-to-high level of agreement (e.g., “strongly agree” or “totally agree”, depending on the response format) in at least *n − 1* items in the respective sections of the questionnaire were classified accordingly. All items were pre-coded so that higher scores systematically reflected more positive responses. To ensure comparability across items characterised by slightly different Likert scales, the response categories were grouped into “agree” versus “neutral/disagree”. The *n − 1* rule was adopted to allow for limited variability in individual responses without underestimating overall competence or positive predisposition, while maintaining the ability to identify differences in knowledge, attitudes, and behaviours. The *n − 1* classification was used only as an operational, exploratory indicator of high endorsement across items and the corresponding regression results should be interpreted as hypothesis-generating rather than confirmatory.

Given the exploratory nature of the study, the non-probabilistic sampling strategy, and the number of potential predictors relative to the sample size, the independent variables were analysed in separate logistic regression models rather than in a single fully adjusted multivariate model. This methodological choice was made in order to reduce the risk of overfitting and unstable parameter estimates, while allowing for an initial exploration of the associations between each independent variable and the outcomes of interest. Each model included only one independent variable at a time (e.g., age, gender, education level, years of professional experience, contract type, access to digital resources, university degree, and postgraduate training), without forcing the inclusion of additional covariates. The results should therefore be interpreted as exploratory and hypothesis-generating rather than confirmatory. Since fully adjusted multivariable models were not fitted, formal multicollinearity diagnostics (e.g., variance inflation factor, VIF) were not computed. Therefore, the reported odds ratios should be interpreted as unadjusted associations and not as independent effects.

All categorical variables were coded using the absence of the characteristic under consideration as the reference category. Consequently, odds ratios (OR) values lower than 1 indicate lower odds of presenting the outcome compared to the reference category. Odds ratios (OR) were calculated with their 95% confidence intervals (95% CI). The level of statistical significance was set at *p* < 0.05. All analyses were performed using SPSS^®^ statistical software version 26.

### 2.6. Ethical Considerations

The study was approved by the Ethics Committee of Sapienza University of Rome with protocol no. 404/20 of 26 March 2020. The questionnaire was preceded by a brief description outlining the details and purpose of the study, to inform the participant while ensuring anonymity and confidentiality of the data. Once the participant had finished reading the study description, they were asked to provide their consent to participate in the study by checking either “yes” or “no.” If the participant did not provide consent to participate in the study and to data processing, they were not able to complete the online questionnaire, and the platform displayed a final “thank you” screen.

## 3. Results

### 3.1. Sociodemographic and Professional Characteristics

The study sample consisted of 320 nurses, with 226 (70.6%) female and 94 (29.4%) male. The mean age was 36.77 years, ranging from 22 to 62 years, with a median of 33.00 years and an interquartile range of 19. The average professional experience was 11.71 years, with a median of 7 years, an interquartile range of 17, and 25th and 75th percentiles of 3 and 20 years, respectively. Geographically, 106 (33.1%) were from the North, 121 (37.8%) from the Center, and 93 (29.1%) from the South of Italy. Information relating to the socio-demographic variables investigated is shown in Table 1.

### 3.2. Descriptive Statistics of Knowledge, Attitudes, and Behavior

Table 2 summarizes the distribution of negative and positive responses for the items included in the knowledge, attitudes, and behaviours domains. Detailed response distributions for each item are provided in the Supplementary file S1.

Overall, participants demonstrated a generally good level of knowledge regarding medication error prevention in home care. Higher levels of agreement were observed for items related to the availability of protocols and informational materials, the provision of personalized therapies, and computerized prescribing and administration systems as elements that can reduce the risk of errors. Conversely, lower levels of adequate knowledge were reported for items related to home care characteristics, workload, and polypharmacy, indicating that these areas are more critical and potentially require targeted educational interventions.

Participants’ attitudes toward safe therapeutic practices were overwhelmingly positive. The vast majority of nurses expressed a favorable attitude toward continuing education, professional awareness, motivation, and adherence to protocols and guidelines. Slightly lower levels of positive attitudes were observed regarding aspects related to nursing prescriptions and managing complex treatment, although overall consensus remained high.

Reported behaviors aimed at preventing medication errors were largely appropriate. High levels of correct behaviors were observed for activities such as medical documentation, adherence to guidelines, pharmacological reconciliation, and monitoring of vital signs. Comparatively lower, though still substantial, adherence was reported for collaboration with pharmacists.

### 3.3. Exploratory Univariate Analyses

For the three domains of the questionnaire (Knowledge, Attitudes, and Behaviors), exploratory univariate analyses were performed at item level. Given the exploratory aim of the study, these item-level analyses were conducted to identify potential patterns across questionnaire items. Therefore, the results should be interpreted as descriptive and hypothesis-generating rather than confirmatory.

Mann–Whitney U tests were used to examine differences between two-group independent variables, whereas Kruskal–Wallis tests were used for independent variables with more than two categories (age, work experience, geographical macro-area, and continuing professional education, grouped into interval classes). When Kruskal–Wallis tests were significant, post hoc pairwise comparisons were conducted using the Mann–Whitney test. Complete Kruskal–Wallis and post hoc outputs are provided in Supplementary file S1, while the main findings are summarized in the text. Because multiple item-level comparisons were performed, these findings should be interpreted with caution.

### 3.4. Exploratory Univariate Analyses for Knowledge

Table 3 presents the results of the univariate analysis, performed using the Mann–Whitney test, to examine differences in mean ranks across population subgroups in relation to the knowledge domain.

With regard to computerized medication prescribing and administration as a potential strategy to prevent medication errors, the Mann–Whitney test shows a statistically significant difference among participants with a university degree (U = 6842.00; Z = −3.283; *p* < 0.05) and among those reporting an excellent level of English language proficiency (U = 9380.00; Z = −2.126; *p* < 0.05). Moreover, participants with a university degree were more likely to agree that intrinsic characteristics of the home care setting (U = 7447; Z = −2.058; *p* < 0.05), an excessive workload (U = 7310; Z = −3.179; *p* < 0.05), and the presence of family members and caregivers (U = 6911.00; Z = −3.144; *p* < 0.05) may represent risk factors for the occurrence of medication errors. No statistically significant differences emerged with respect to the presence or absence of specific content on home medication management during basic training; however, the Mann–Whitney test was significant regarding specific content in post-basic courses. Specifically, participants who had not received specific training on home medication management were more likely to agree that an excessive workload (U = 11,594; Z = −2.001; *p* < 0.05) and the presence of family members and caregivers in the care setting (U = 11,203; Z = −2.110; *p* < 0.05) are risk factors for the occurrence of medication therapy errors. Finally, those reporting an excellent level of English language proficiency were more likely to agree that an excessive workload (U = 9160; Z = −3.239; *p* < 0.05) represents a potential risk for the occurrence of medication errors.

For the knowledge domain, statistically significant Kruskal–Wallis tests were followed by post hoc pairwise comparisons (Mann–Whitney). Full results are reported in Supplementary file S1 (Tables S4–S10), while the main findings are summarized below.

Statistically significant post hoc differences were observed for the item on computerized prescribing and administration across work-experience categories, including 0–5 vs. 21–25 years (U = 1505.00; Z = −2.217; *p* = 0.027) and 6–10 vs. 21–25 years (U = 742.00; Z = −2.058; *p* = 0.040). A significant post hoc difference was also observed across weekly continuing professional education, between 1–5 h/week vs. >11 h/week (U = 53.00; Z = −2.146; *p* = 0.032). No statistically significant post hoc differences were observed for age or geographical macro-area (Table S4).

For the item on an individualized medication supply system, a statistically significant post hoc difference was observed across geographical macro-areas, between Northern and Central Italy (U = 5578.50; Z = −2.159; *p* = 0.031). No statistically significant post hoc differences were observed for age, work experience, or weekly continuing professional education (Table S5).

No statistically significant post hoc differences were observed for the item on the use of protocols/information brochures in specific situations or for the item on intrinsic characteristics of the home care setting as a risk factor, across age, work experience, geographical macro-area, and weekly continuing professional education (Tables S6 and S7).

Statistically significant post hoc differences were observed for the item on excessive workload as a risk factor, across both age (e.g., 20–25 vs. 56–60 years: U = 243.00; Z = −2.260; *p* = 0.024; and 26–30 vs. 56–60 years: U = 366.50; Z = −2.450; *p* = 0.014) and work-experience categories (e.g., 0–5 vs. 36–40 years: U = 25.50; Z = −4.273; *p* < 0.001; and 16–20 vs. 36–40 years: U = 0.00; Z = −4.472; *p* < 0.001). Full pairwise comparisons are provided in Supplementary file S1 (Table S8).

For the item on the presence of family members/caregivers as a potential source of distraction, statistically significant post hoc differences were observed across age (e.g., 20–25 vs. 51–55 years: U = 578.00; Z = −3.184; *p* = 0.001; and 31–35 vs. 51–55 years: U = 694.00; Z = −2.728; *p* = 0.006) and work experience (e.g., 0–5 vs. 36–40 years: U = 51.00; Z = −2.911; *p* = 0.004). No statistically significant post hoc differences were observed for geographical macro-area or weekly continuing professional education. Full pairwise comparisons are provided in Supplementary file S1 (Table S9).

Finally, for the item on polypharmacy as a risk factor, a statistically significant post hoc difference was observed across geographical macro-areas between Northern and Southern Italy (U = 4237.50; Z = −2.032; *p* = 0.042). No statistically significant post-hoc differences were observed for age, work experience, or weekly continuing professional education (Table S10).

### 3.5. Exploratory Univariate Analyses for Attitudes

Table 4 presents the results of the univariate analysis, performed using the Mann–Whitney test, to examine differences in mean ranks across population subgroups in relation to the attitudes domain.

Regarding nurses’ prescribing of medications and/or aids as an action that may contribute to improving medication management in home care, the Mann–Whitney test shows a statistically significant difference among participants who had completed post-basic training (U = 11,198.50; Z = −2.338; *p* < 0.05) and among those who had received specific content on home medication management during post-basic training (U = 11,310.50; Z = −1.995; *p* < 0.05). In addition, participants who had not received specific content on home medication management during basic training were more likely to agree that operator motivation can improve the performance of the overall medication-management process (U = 10,700.00; Z = −2.286; *p* < 0.05). Finally, participants reporting an insufficient level of English language proficiency were more likely to agree that respecting infusion rates reduces the risk of medication errors compared with those reporting excellent proficiency (U = 10,120.00; Z = −2.050; *p* < 0.05). Workplace internet access was also associated with Att_4, with a statistically significant difference between those with and without internet access at work (U = 3649.00; Z = −3.751; *p* < 0.001).

Tables S11–S17 (Supplementary file S1) report the results of the Kruskal–Wallis tests and the post hoc pairwise comparisons performed using the Mann–Whitney test, in relation to categorical independent variables such as age, work experience, geographical macro-area, and continuing professional education. The dependent variable is represented by each of the seven items belonging to the attitudes domain; the independent variables were divided into interval classes. The main findings are presented in the text, while details of the comparisons can be consulted in the tables in Supplementary file S1.

Statistically significant post hoc differences by geographical macro-area were observed between Northern and Central Italy (U = 5579.50; Z = −2.699; *p* = 0.007; mean ranks 121.86 vs. 107.11) and between Northern and Southern Italy (U = 4446.50; Z = −2.039; *p* = 0.041; mean ranks 104.55 vs. 94.81). No statistically significant differences were observed for age, work experience, or weekly continuing professional education (Table S11).

Statistically significant post hoc differences were observed by weekly continuing professional education: <1 h/week vs. 1–5 h/week (U = 9902.50; Z = −1.970; *p* = 0.049; mean ranks 143.21 vs. 153.68) and 1–5 h/week vs. 6–10 h/week (U = 1472.50; Z = −2.616; *p* = 0.009; mean ranks 101.77 vs. 84.13) (Table S12). No statistically significant differences were observed for age, work experience, or geographical macro-area.

A statistically significant post hoc difference was observed by weekly continuing professional education: <1 h/week vs. 6–10 h/week (U = 983.00; Z = −2.003; *p* = 0.045; mean ranks 71.74 vs. 59.65) (Table S14). No statistically significant differences were observed for age, work experience, or geographical macro-area.

Tables S13 and S15–S17 report that no statistically significant post hoc differences were observed for the items assessing nurses’ motivation, nurse prescribing, infusion rate, and the “8 rights” rule, across age, work experience, geographical macro-area, and weekly continuing professional education.

### 3.6. Exploratory Univariate Analyses for Behaviours

Table 5 presents the results of the univariate analysis, performed using the Mann–Whitney test, to examine differences in mean ranks across population subgroups in relation to the behaviors domain. Statistically significant differences were observed for educational pathway and workplace internet access. Nurses with a university degree reported higher agreement with the statement that monitoring vital signs before and after intravenous medication administration reduces complications and improves medication management in home care (U = 7319.50; Z = −3.437; *p* = 0.001). In addition, nurses with internet access at work reported higher agreement with the statement that, when taking charge of a new patient, having comprehensive documentation for medication reconciliation reduces errors throughout the medication-management process (U = 3741.00; Z = −3.585; *p* < 0.001). No other statistically significant differences were observed in the Mann–Whitney comparisons (Table 5).

Tables S18–S23 (Supplementary file S1) report the results of Kruskal–Wallis tests and post hoc pairwise comparisons performed using the Mann–Whitney test, in relation to categorical independent variables such as age, work experience, geographical macro-area, and continuing professional education. The dependent variables were the six items belonging to the behaviors domain, while the independent variables were grouped into interval classes. The main findings are presented in the text, whereas full pairwise comparison details are provided in Supplementary file S1.

Post hoc comparisons showed statistically significant differences by age for the item assessing collaboration with the pharmacist: older age groups reported higher agreement than participants aged 20–25 years (20–25 vs. 31–35 years: U = 1012.50; Z = −3.214; *p* = 0.001; 20–25 vs. 36–40 years: U = 334.50; Z = −1.966; *p* = 0.049; 20–25 vs. 46–50 years: U = 729.00; Z = −2.519; *p* = 0.012; 20–25 vs. 51–55 years: U = 569.50; Z = −3.130; *p* = 0.002; 20–25 vs. 56–60 years: U = 226.00; Z = −2.034; *p* = 0.042) (Table S18).

For the item assessing health documentation, statistically significant post hoc differences by age indicated higher agreement among nurses aged 41–45 years compared with younger groups (20–25 vs. 41–45 years: U = 616.00; Z = −2.040; *p* = 0.041; 26–30 vs. 41–45 years: U = 924.00; Z = −2.104; *p* = 0.035) (Table S19).

Geographical macro-area differences emerged for the item assessing support guidelines, with higher agreement among nurses working in Northern Italy compared with those working in Central Italy (U = 5700.50; Z = −2.256; *p* = 0.024) (Table S20).

For the item assessing medication reconciliation, statistically significant post hoc differences were observed by weekly continuing professional education (<1 h/week vs. 1–5 h/week: U = 9603.00; Z = −2.310; *p* = 0.020) and by geographical macro-area (Central vs. Southern Italy: U = 5081.50; Z = −1.965; *p* = 0.049) (Table S21).

For the item assessing use of hydroalcoholic gel, statistically significant post hoc differences were found by geographical macro-area (Central vs. Southern Italy: U = 4799.00; Z = −3.204; *p* = 0.001) and by weekly continuing professional education (6–10 h/week vs. >11 h/week: U = 11.00; Z = −2.062; *p* = 0.039) (Table S22).

Finally, for the item assessing vital signs monitoring, post hoc comparisons showed lower agreement among nurses with 31–35 years of work experience compared with less-experienced groups (0–5 vs. 31–35 years: U = 952.00; Z = −3.733; *p* = 0.001; 6–10 vs. 31–35 years: U = 478.00; Z = −3.088; *p* = 0.002; 11–15 vs. 31–35 years: U = 128.00; Z = −1.993; *p* = 0.046; 16–20 vs. 31–35 years: U = 118.00; Z = −2.461; *p* = 0.014; 21–25 vs. 31–35 years: U = 208.00; Z = −2.007; *p* = 0.045). Geographical macro-area differences were also observed, with higher agreement in Central and Southern Italy compared with Northern Italy (Northern vs. Central Italy: U = 5657.50; Z = −2.447; *p* = 0.014; Northern vs. Southern Italy: U = 4124.50; Z = −3.295; *p* = 0.001) (Table S23).

### 3.7. Exploratory Logistic Regression Model on the National Sample

Using the exploratory dichotomous indicators derived from the “n − 1” rule, separate logistic regression models were fitted for each predictor (Table 6). As described in the Material and methods section, these models were intentionally specified as unadjusted exploratory models in order to avoid overfitting given the sample size and the number of potential predictors. Findings should therefore be interpreted as hypothesis-generating rather than confirmatory.

Regarding the knowledge domain, the results show a statistically significant difference in relation to age and educational level (Table 6). Specifically, nursing professionals with a non-university degree are significantly more likely to demonstrate adequate knowledge compared to those with a university degree (OR: 0.43, 95% CI: 0.23–0.77; *p* < 0.001). Age shows a marginally significant trend, with younger professionals slightly more likely to have adequate knowledge (OR: 0.97, 95% CI: 0.95–1.00; *p* = 0.050). No significant associations were found for sex, years of work experience, completion of post-basic training, or having received specific education on medication management during basic or post-basic courses.

For the attitudes domain, only access to the internet at the workplace was significantly associated with outcomes (Table 6). Nurses with internet access at their workstation were less likely to report positive attitudes compared to those without access (OR: 0.41, 95% CI: 0.19–0.86, *p* < 0.05). All other variables, including sex, age, education, work experience, access to library resources, and English proficiency, were not significantly associated with attitudes.

Similarly, for the behaviors domain, internet access at the workplace was the only variable showing a significant association (Table 6). Nurses with internet access were less likely to demonstrate correct behaviors (OR: 0.45, 95% CI: 0.21–0.96, *p* < 0.05). No other socio-demographic or professional characteristics were significantly associated with behaviors.

Following the model proposed by Di Muzio et al. [20], logistic regression analyses were conducted to examine the relationships among knowledge, attitudes, and behaviors (Table 7). Descriptive analysis shows that 84% of participants categorized as having adequate knowledge also exhibit positive attitudes. Inferentially, nurses with negative attitudes toward medication safety are more than twice as likely to demonstrate inadequate behaviors compared to those with positive attitudes (OR: 2.22, 95% CI: 1.29–3.96, *p* < 0.005).

This is also confirmed by the correlation analyses (Spearman’s rho), which show significant positive associations among all three domains: knowledge with attitudes (ρ = 0.162; *p* = 0.004), knowledge with behaviors (ρ = 0.166; *p* = 0.003), and attitudes with behaviors (ρ = 0.367; *p* < 0.001) (Table 8). Notably, the strongest association was observed between attitudes and behaviors, reinforcing the role of positive attitudes as a key factor influencing safe practices.

## 4. Discussion

This study aimed to investigate factors associated with nurses’ knowledge, attitudes, and self-reported behaviours regarding strategies to prevent medication errors in the Italian home care setting.

The findings highlight the interdependence of these three domains and suggest that both individual and organisational factors may influence safe medication management practices in home care. These results are consistent with previous studies exploring medication safety through the KAB framework, which have shown that nurses’ knowledge, attitudes, and behaviours are closely interconnected and jointly contribute to safer clinical practices. Earlier investigations conducted in hospital settings, particularly in intensive care units, have demonstrated similar relationships between these domains in relation to medication error prevention [14].

The questionnaire used in the present study derives from this line of research and was subsequently adapted and validated for the home care context in Italy and later in Spain, allowing the investigation of medication safety dimensions in community-based care settings [17,18].

Indeed, the international scientific literature divides risk factors into two main areas: those related to the personal and professional characteristics of healthcare professionals and those related to the organization of the complex process of medication management [3,15,20]. Considering the specific context of the research, the literature highlights that medication errors are among the three main causes of adverse or potentially harmful events for individuals [22,23,24,25]. Based on these considerations, as well as previous national and international [14] studies, this research sought to investigate the correlation between etiological factors that may contribute to harmful or potentially harmful events for the patient, with a focus on variables related to the nursing professional. The results obtained from this study suggest a relationship between positive attitudes and correct behavior and/or adequate knowledge, as well as between adequate knowledge and correct behavior, in line with the results of previous national and international studies.

Careful consideration must be given to the factor associated with errors related to the work environment. Environmental and organizational factors remain relevant contributors to medication errors in home care [24,26]. In this sample, polypharmacy was frequently endorsed as a potential risk factor, reflecting the clinical complexity of patients receiving home care and the challenges associated with medication management in this setting [27,28,29]. These findings are consistent with previous literature highlighting the role of workload, context characteristics, and patient complexity in increasing the risk of medication errors in home healthcare [12,30]. However, compared with hospital settings, medication management in home care occurs in less standardized environments and often involves additional actors such as family members or informal caregivers [12]. These contextual characteristics may increase the cognitive and organisational demands placed on nurses, potentially amplifying the impact of factors such as polypharmacy, interruptions, and communication difficulties [2]. For this reason, medication safety strategies developed for hospital environments may require adaptation when applied to home care settings [3]. These findings should also be interpreted in light of the Italian healthcare system, where home care services are organised at the regional level and may differ in terms of organisational models, available resources, and professional roles.

Differences across selected sociodemographic and professional characteristics were observed for several preventive strategies related to medication safety. Polypharmacy was frequently recognised as a potential risk factor, which is consistent with previous literature identifying polypharmacy as a major contributor to medication errors in complex care settings. Assiri et al. [31] identified polytherapy as one of the primary causes of medication errors, defining it as a risk factor both for the patient and for the professional. Given that this variable is not easily eliminated, due to the socio-health conditions of most of the population receiving home care, preventive strategies become crucial. Literature suggests pharmacological reconciliation, collaboration with pharmacists, and the use of computerized documentation as preventive strategies. In this regard, significant space is given in the literature to the use of computerized systems for medication prescription and administration to prevent errors, both in hospital settings and in home healthcare [29,32]. These strategies may be particularly relevant in the home care setting, where medication management frequently involves multiple prescribers and fragmented information flows between healthcare providers. In such contexts, tools that support medication reconciliation, shared documentation, and interprofessional collaboration may play a crucial role in reducing inconsistencies in prescriptions and improving the overall safety of medication management [29].

The role of the pharmacist and the possibility of individualized medication delivery systems are defined by the national sample as possible preventive strategies for medication errors. Several studies have evaluated the impact of introducing pharmacists into home care teams to improve continuity of care, documenting a reduction in medication errors and improvements in the medication reconciliation process [33,34].

The finding that nurses without a university degree were more likely to demonstrate adequate knowledge compared to graduates contrasts with part of the international literature [35,36]. This finding should be interpreted cautiously and may reflect unmeasured differences between qualification groups (e.g., roles, organisational context, or training exposure), which were not captured in the present dataset. However, given the exploratory unadjusted modelling strategy, residual confounding cannot be excluded. In the Italian context, many nurses without a university degree obtained their qualification before the introduction of the university-based nursing education system and may therefore have accumulated longer professional experience in community or home care settings. In addition, it is possible that nurses with longer professional trajectories or greater familiarity with home care practice may have developed experiential knowledge related to medication management in community settings. These findings highlight the importance of continuous professional development for nurses across different educational backgrounds.

In the exploratory analyses, years of work experience were not consistently associated with the outcomes, suggesting that experience alone may be insufficient to ensure sustained knowledge and safe practices. Similar findings have been reported in previous studies investigating medication safety, where professional experience alone did not necessarily translate into higher adherence to safe medication practices. This underscores the need for continuous professional development programs to keep nursing staff up to date with evolving clinical practices.

Positive attitudes of nurses also play a fundamental role in the occurrence of medication errors. Attitudes can be conceptualised as predispositions that may influence how individuals interpret information and translate knowledge into practice. In this study, attitudes showed the strongest association with behaviours, supporting the relevance of motivational and culture-of-safety components in educational interventions [37,38]. These considerations are consistent with the findings in the attitude section of the questionnaire.

Contrary to expectations, workplace internet access was negatively associated with positive attitudes and correct behaviours. This finding should be interpreted cautiously given the cross-sectional design and the imbalance between groups (a small proportion reported no internet access). Rather than indicating a direct causal effect, it may suggest that access alone is insufficient, or that unmeasured organisational factors (e.g., workload, digital governance, and training on the effective use of evidence-based online resources) may be involved. Therefore, this finding should be interpreted cautiously and may reflect contextual or organisational characteristics rather than a direct effect of internet availability.

Similarly, nurses with internet access at work were also less likely to demonstrate correct behaviors. This association should not be interpreted as evidence that internet access worsens safety-related behaviours. Instead, it highlights the need for future studies to examine how digital resources are implemented and used in home care practice, including training, governance, and workflow integration. Methodological considerations are warranted. The dichotomous outcomes were defined through an operational “n − 1” rule and should not be interpreted as validated thresholds of competence. Dichotomization may entail information loss and misclassification; therefore, we intentionally restrict inference from these models and interpret them as exploratory signals that need confirmation in future studies using approaches that retain the full information of the scores.

The logistic regression analyses further support the interdependence between knowledge, attitudes, and behaviours, suggesting that motivational and cognitive components jointly influence safety-related practices. In line with the model proposed by Di Muzio et al. [14], positive attitudes were found to be a significant factor associated with adequate knowledge, reinforcing the idea that motivation and openness to learning play a crucial role in knowledge acquisition. Additionally, the results indicate that both knowledge and positive attitudes contribute to better behavioral outcomes, emphasizing the need for a holistic approach in nursing education that integrates cognitive learning with attitude-forming strategies. These results reinforce the importance of considering cognitive, motivational, and behavioral aspects as a whole when designing training and professional development programs aimed at reducing medication errors.

The correlation analyses revealed statistically significant, albeit weak to moderate, associations among knowledge, attitudes, and behaviours. While these correlations do not imply causality, they support the internal coherence of the KAB framework by showing consistent directional relationships among the three domains at score level. Unlike the logistic regression analyses, which explored associations using dichotomised outcomes, the Spearman correlations retained the ordinal nature of the data and therefore provide complementary information on the overall pattern of relationships. In particular, the stronger association observed between attitudes and behaviours reinforces the conceptual role of attitudes as a potential bridge between knowledge and practice, without overlapping with the exploratory regression findings.

### Limitations

This study is, to the best of our knowledge, the first national research investigating medication error prevention in the home care setting. However, it is not without limitations.

First, convenience sampling may have introduced selection bias and limits the generalizability of the results. Furthermore, it was not possible to make an a priori estimate of the sample size, as there is no national database reporting the total number of nurses employed in home care in Italy. Although participants came from diverse geographic regions, potential regional heterogeneity was not specifically analyzed; differences in organizational models, resource availability, and local policies may have influenced the observed results. Furthermore, although participants were recruited from the three main geographical macro-areas of Italy (North, Center, and South), the distribution of respondents across regions may not reflect the actual distribution of nursing staff working in home care in Italy.

Second, data collection via Google Forms^®^ and the dissemination of the questionnaire through online channels and social media did not allow for a response rate calculation or an accurate estimate of the number of potential participants reached. For the same reason, it was not possible to guarantee with certainty that each participant completed the questionnaire only once: although no obvious duplications were identified during data cleaning, the possibility of multiple responses cannot be completely ruled out. Furthermore, the online recruitment method may have favored the participation of younger participants, those with greater digital familiarity and/or greater interest in the topic, further reducing the representativeness of the sample [39,40]. Since baseline information on the national population was not available, it was not possible to apply sample weights; therefore, the results should not be interpreted as nationally representative, but rather as exploratory and hypothesis-generating tests.

An additional limitation concerns the self-report nature of the instrument: responses may be influenced by distractions during completion and social desirability bias, and the information reported was not independently verifiable, potentially risking reporting bias [41]. Furthermore, eligibility criteria were assessed through self-reported screening questions, which may have introduced a limited risk of misclassification.

Regarding the analyses, the cross-sectional design does not allow for causal inferences. Furthermore, the regression strategy adopted (separate logistic models for each predictor) does not allow for the joint effect of the variables to be assessed or adequately controlled for confounding among correlated predictors; consequently, some associations may reflect spurious relationships. Running multiple models also increases the risk of results due to chance (Type I error inflation). Finally, dichotomizing outcomes using the n − 1 criterion, while useful for exploratory analysis, results in a loss of information and reduced variability; therefore, the results should be interpreted with particular caution and consideration should be given to hypotheses to be tested in future studies using more robust analytical strategies.

A significant limitation is that medication errors were not directly measured as an outcome. Direct measurement of medication errors in home care settings presents several methodological challenges, including underreporting, variability in reporting systems, and the absence of standardized monitoring mechanisms across services. For this reason, the present study focused on indirect determinants of medication safety, such as nurses’ knowledge, attitudes, and behaviours related to medication management. These dimensions represent modifiable factors that may influence clinical practice and are frequently used in safety research to explore professional preparedness and preventive strategies in complex care settings. Accordingly, the analysis focused on knowledge, attitudes, and behaviours as indirect dimensions associated with safer medication management practices. Therefore, the results should not be interpreted as factors directly associated with medication errors, but rather as factors associated with professional behaviours and dispositions potentially conducive to medication safety.

Finally, the study’s timeframe (during the COVID-19 pandemic) may have influenced perceptions, attitudes, and self-reported behaviors, in a context characterized by increased workload, stress, and organizational changes. Therefore, the results should also be interpreted in light of this exceptional context.

## 5. Conclusions

This study provides insights into the factors influencing medication error prevention in the home care setting, particularly focusing on the knowledge, attitudes, and behaviors of nurses.

In this sample, adequate knowledge was more frequently observed among nurses without a university degree; age showed only a borderline association with the knowledge outcome. The lack of a significant association between knowledge and years of work experience further emphasizes the need for continuous professional development to ensure that nursing staff remain up to date with evolving clinical practices.

Moreover, positive attitudes were found to be a significant factor associated with both adequate knowledge and appropriate behaviors, highlighting the importance of fostering a positive mindset among healthcare professionals.

However, contrary to expectations, internet access at the workplace was negatively associated with both attitudes and behaviors, suggesting that the relationship between access to digital resources and safety-related attitudes/behaviours may be influenced by unmeasured contextual and organisational factors. This finding underscores the importance of promoting not only access to information but also the skills and organizational support needed to use digital tools effectively in clinical decision-making.

The results also emphasize the complexity of medication management in home care, where factors such as polytherapy and the lack of therapeutic surveillance increase the risk of medication errors. The study identifies the need for preventive strategies, such as collaboration with pharmacists and the use of computerized systems for medication prescribing and administration, which are commonly recommended to support medication safety and medication reconciliation in home care settings.

Future research should address the limitations of this study and explore additional environmental and organizational factors that may influence medication safety in home care. Therefore, future research using longitudinal approaches and probability sampling is needed to further explore medication safety in home care.

### Implication for Practice

The findings of this study suggest several practical implications for nursing practice in the home care setting.

First, the observed associations between knowledge, attitudes, and behaviors highlight the importance of training strategies that address not only technical knowledge but also behaviors related to medication safety. Training programs should therefore adopt an integrated approach that promotes reflective practice, professional accountability, and adherence to evidence-based guidelines, providing access to appropriate resources and supporting ongoing professional development. In particular, nursing education in the home care setting should place greater emphasis on medication reconciliation, polypharmacy management, communication with caregivers and general practitioners, and the effective use of digital tools to support safe medication practices. Educational strategies should also address attitudinal components, such as safety culture, professional awareness, and motivation, since these dimensions may influence the translation of knowledge into practice.

Second, polypharmacy and the complexity of the home care context as significant risk factors underscore the need for structured preventive strategies, such as medication reconciliation processes, standardized documentation, and interprofessional collaboration, particularly with pharmacists.

Finally, the findings regarding access to digital resources indicate that the availability of technological tools alone may not be sufficient to promote safe practices. Healthcare organizations should therefore ensure that nurses are adequately supported through targeted training and organizational policies that facilitate the effective and appropriate use of digital resources in the clinical decision-making process.

## Figures and Tables

**Table 1:** Demographics and professional characteristics of the nursing sample.

Variable	n (%)
Gender (Male vs. Female)	94 (29.4)/226 (70.6)
Age	36.77 (33; 19) *
Work experience in years	11.71 (7; 17; 3 and 20) **
University degree (university degree/no-university degree)	251 (78.4)/69 (21.6)
Post-degree education (post-degree education vs. none post degree education)	149 (46.6)/171 (53.4)
Training on the management of pharmacological therapy in the home setting during undergraduate education (Yes vs. No)	118 (36.9)/202 (63.1)
Training on the management of pharmacological therapy in the home setting during post-graduate education (Yes vs. No)	141 (44.1)/179 (55.9)
Access to Internet connection at the workplace (Yes vs. No)	286 (89.4)/34 (10.6)
Access to library at the workplace (Yes vs. No)	127 (39.7)/193 (60.3)
Knowledge of English language (insufficient vs. excellent)	225 (70.3)/95 (29.7)
Hours per week to continuous education	
<2 h per week	119 (37.2)
2–5 h per week	179 (55.9)
6–10 h per week	20 (6.3)
≥11 h per week	2 (0.6)

* median; interquartile range. ** median; interquartile range; 25th and 75th percentile.

**Table 2:** Distribution of negative and positive responses for knowledge, attitudes, and behaviours items.

Domain	Item	Negative Responses n (%)	Positive Responses n (%)
***Knowledge***	Computerized prescription and administration	105(32.8)	215 (67.2)
	Individualized therapy supply	92 (28.7)	228 (71.3)
	Protocols and informational brochures	61 (19.1)	259 (80.9)
	Characteristics of the home setting	155 (48.4)	165 (51.6)
	Workload	49 (15.3)	174 (54.4)
	Presence of family members and caregivers	107 (33.4)	213 (66.6)
	Polypharmacy	128 (40.0)	192 (60.0)
***Attitudes***	Continuing education	52 (16.3)	268 (83.8)
	Professional awareness	35 (10.9)	285 (89.1)
	Professional motivation	58 (18.1)	262 (81.9)
	Protocols, guidelines, and procedures	51 (15.9)	269 (84.1)
	Nursing prescription	98 (30.6)	222 (69.4)
	Infusion speed	15 (4.7)	305 (95.3)
	“8 rights” rule	13 (4.1)	307 (95.9)
***Behaviours***	Collaboration with pharmacist	109 (34.1)	211 (65.9)
	Medical documentation	27 (8.4)	293 (91.6)
	Support guidelines	48 (15.0)	272 (85.0)
	Medication reconciliation	47 (14.7)	273 (85.3)
	Use of hydroalcoholic gel	38 (11.9)	282 (88.1)
	Monitoring vital signs	40 (12.5)	280 (87.5)

Negative responses correspond to “strongly disagree”, “disagree” and “neutral” for knowledge and behaviour items, “disagree” and “neutral” for attitude items. Positive responses correspond to “agree” or “strongly agree” for knowledge and behaviour items, “agree” for attitude items.

**Table 3:** Results of comparisons made using the Mann–Whitney test: sociodemographic characteristics and knowledge.

Variable		Know_1	Know_2	Know_3	Know_4	Know_5	Know_6	Know_7
Sex	U	9847	9558	10,155	10,057	10,365	10,051	10,046
	Z	−1.264	−1.631	−0.911	−0.866	−0.547	−0.927	−0.900
	*p*	0.206	0.103	0.363	0.387	0.585	0.354	0.368
*Male*	MR	168.74	170.76	165.47	166.51	155.77	154.43	166.63
*Female*	MR	157.07	156.23	158.43	158.00	161.64	163.03	157.95
Title of study	U	6842.00	8473.50	7884.00	7447.00	7310.00	6911.00	8115.50
	Z	−3.283	−0.349	−1.675	−2.058	−3.179	−3.144	−0.942
	*p*	0.001 *	0.727	0.094	0.040 *	0.001 *	0.002 *	0.346
*University*	MR	167.74	161.24	163.59	165.33	165.88	167.47	162.67
*Non university*	MR	134.16	157.80	149.26	142.93	140.94	135.16	152.62
Education post-base	U	12,242.00	12,553.50	12,164.00	12,607.00	11,489.00	12,288.00	12,163.50
	Z	−0.741	−0.287	−1.025	−0.185	−2.428	−0.669	−0.822
	*p*	0.459	0.774	0.306	0.853	0.015 *	0.503	0.411
*No*	MR	163.41	161.59	16.87	161.27	153.19	157.86	157.13
*Yes*	MR	157.16	159.25	156.64	159.61	168.89	163.53	164.37
Medication errors argument in university courses	U	11,393.00	11,770.00	11,357.00	11,623.00	11,289.00	11,671.00	11,630.00
	Z	−0.808	−0.236	−1.033	−0.427	−1.263	−0.379	−0.425
	*p*	0.419	0.813	0.302	0.670	0.207	0.705	0.671
*No*	MR	163.10	159.77	163.28	161.96	163.61	161.72	161.93
*Yes*	MR	156.05	161.75	155.75	158.00	155.17	158.41	158.06
Medication errors argument in post university courses	U	12,182.00	12,213.50	12,479.00	12,027.00	11,594.00	11,203.00	11,883.50
	Z	−0.655	−0.630	−0.251	−0.833	−2.001	−2.110	−1.056
	*p*	0.513	0.528	0.802	0.405	0.045 *	0.035 *	0.291
*No*	MR	162.94	158.23	159.72	163.81	166.23	168.41	164.61
*Yes*	MR	157.40	163.38	161.50	156.30	153.23	150.45	155.28
Level of english language knowledge	U	9380.00	10,417.50	10,385.00	9725.00	9160.00	9925.00	10,527.50
	Z	−2.126	−0.455	−0.588	−1.471	−3.239	−1.234	−0.249
	*p*	0.033 *	0.649	0.557	0.141	0.001 *	0.217	0.803
*Insufficient*	MR	154.69	161.70	161.84	156.22	153.71	157.11	159.79
*Excellent*	MR	174.26	157.66	157.32	170.63	176.58	168.53	162.18
Access to Internet connection	U	4357.00	4666.00	4779.00	4777.00	4669.00	4601.00	4318.00
	Z	−1.218	−0.490	−0.239	−0.193	−0.607	−0.626	−1.257
	*p*	0.223	0.624	0.811	0.847	0.544	0.531	0.209
*No*	MR	175.35	154.74	158.06	158.00	166.18	152.82	144.50
*Yes*	MR	158.73	161.19	160.79	160.80	159.83	161.41	162.40
Access to library	U	11,668.00	11,693.50	12,129.00	11,218.00	11,864.00	11,701.00	11,743.50
	Z	−0.892	−0.885	−0.230	−1.480	−0.775	−0.838	−0.745
	*p*	0.372	0.376	0.818	0.139	0.438	0.402	0.456
*No*	MR	157.46	157.59	161.16	165.88	158.47	157.63	163.15
*Yes*	MR	165.13	164.93	159.50	152.33	163.58	164.87	156.47

U: U di Mann–Whitney; MR: Mean Rank; * statistically significant value. Know_1: computerized prescription and administration. Know_2: individualized therapy provision. Know_3: protocols and information brochures. Know_4: characteristics of the home setting. Know_5: workload. Know_6: presence of family members and caregivers. Know_7: polytherapy.

**Table 4:** Results of comparisons made using the Mann–Whitney test: sociodemographic characteristics and attitude.

Variable		Att_1	Att_2	Att_3	Att_4	Att_5	Att_6	Att_7
Sex	U	9866.00	10,507.00	9988.00	10,619.00	10,428.00	10,527.00	10,331.0
	Z	−1.569	−0.282	−1.260	−0.006	−0.322	−0.344	−1.129
	*p*	0.117	0.778	0.208	0.995	0.747	0.731	0.259
*Male*	MR	152.46	159.28	153.76	160.47	158.44	159.49	163.60
*Female*	MR	163.85	161.01	163.31	160.51	161.36	160.92	159.21
Title of study	U	8373.50	8412.00	8100.50	8500.00	8158.50	8377.00	8626.00
	Z	−0.658	−0.673	−1.231	−0.370	−0.922	−1.134	−0.135
	*p*	0.511	0.501	0.218	0.712	0.357	0.257	0.892
*University*	MR	161.64	159.51	162.73	159.86	158.50	161.63	160.63
*Non university*	MR	156.36	164.09	152.40	162.81	167.76	156.41	160.04
Education post-base	U	12,545.5	12,467.00	11,938.50	12,460.00	11,198.50	12,097.00	12,428.00
	Z	−0.368	−0.611	−1.454	−0.534	−2.338	−2.126	−1.103
	*p*	0.713	0.541	0.146	0.593	0.019 *	0.034 *	0.270
*No*	MR	159.37	162.09	155.82	158.87	151.49	164.26	162.32
*Yes*	MR	161.80	158.67	165.88	162.38	170.84	156.19	158.41
Medication errors argument in university courses	U	11,786.0	11,583.00	10,700.00	11,407.00	11,736.00	11,673.00	11,885.00
	Z	−0.259	−0.776	−2.286	−1.009	−0.285	−0.838	−0.121
	*p*	0.796	0.438	0.022 *	0.313	0.775	0.402	0.904
*No*	MR	161.15	162.16	166.53	163.03	159.60	159.29	160.66
*Yes*	MR	159.38	157.66	150.18	156.17	162.04	162.58	160.22
Medication errors argument in post university courses	U	12,605.5	12,232.00	12,050.50	12,375.00	11,310.50	12,362.00	12,343.00
	Z	−0.027	−0.872	−1.038	−0.469	−1.995	−0.856	−0.984
	*p*	0.979	0.383	0.299	0.639	0.046 *	0.392	0.325
*No*	MR	160.58	158.34	157.32	161.87	153.19	159.06	158.96
*Yes*	MR	160.40	163.25	164.54	158.77	169.78	162.33	162.46
Level of english language knowledge	U	10,137.5	10,270.00	10,402.50	10,390.00	10,512.50	10,120.00	10,505.00
	Z	−1.138	−1.021	−0.565	−0.621	−0.290	−2.050	−0.706
	*p*	0.255	0.307	0.572	0.535	0.772	0.040 *	0.480
*Insufficient*	MR	158.06	162.36	161.77	161.82	159.72	163.02	161.31
*Excellent*	MR	166.29	156.11	157.50	157.37	162.34	154.53	158.58
Internet connection	U	4306.00	4657.00	4728.00	3649.00	4128.00	4676.00	4763.00
	Z	−1.706	−0.744	−0.394	−3.751	−1.803	−0.509	−0.568
	*p*	0.088	0.457	0.694	<0.001 *	0.071	0.611	0.570
*No*	MR	144.15	154.47	156.56	124.82	138.91	163.29	157.59
*Yes*	MR	162.44	161.22	160.97	164.74	163.07	160.17	160.85
Access to library	U	12,153.5	12,238.00	12,092.50	12,057.00	11,918.50	11,943.00	12,070.00
	Z	−0.197	−0.040	−0.302	−0.387	−0.521	−1.054	−0.670
	*p*	0.844	0.968	0.763	0.699	0.602	0.292	0.503
*No*	MR	159.97	160.59	159.66	159.47	162.25	158.88	159.54
*Yes*	MR	161.30	160.36	161.78	162.06	157.85	162.96	161.96

U: U di Mann–Whitney; MR: Medium Rank; * statistically significant value. Att_1: continuing education. Att_2: nurses’ professional awareness. Att_3: nurses’ motivation. Att_4: protocols, guidelines, and procedures. Att_5: nurse prescribing. Att_6: infusion rate. Att_7: the “8 rights” rule.

**Table 5:** Results of comparisons made using the Mann–Whitney test: sociodemographic characteristics and behaviours.

Variable		Behav_1	Behav_2	Behav_3	Behav_4	Behav_5	Behav_6
Sex	U	10,139.00	10,131.00	9838.00	10,111.00	10,276.00	10,502.00
	Z	−0.781	−1.353	−1.682	−1.106	−0.819	−0.278
	*p*	0.435	0.176	0.093	0.269	0.413	0.781
*Male*	MR	165.64	055.28	152.16	155.06	164.18	161.78
*Female*	MR	158.36	162.67	163.97	162.76	158.97	159.97
Title of study	U	7779.00	8048.00	8603.50	8478.00	8370.50	7319.50
	Z	−1.576	−1.866	−0.133	−0.435	−0.758	−3.437
	*p*	0.115	0.062	0.894	0.664	0.449	0.001 *
*University*	MR	156.99	158.06	160.28	159.78	161.65	165.84
*Non university*	MR	173.26	169.36	161.31	163.13	156.31	141.08
Education post-base	U	12,299.00	12,008.00	12,363.50	12,598.00	12,628.50	12,519.50
	Z	−0.650	−1.840	−0.736	−0.280	−0.240	−0.465
	*p*	0.516	0.066	0.461	0.780	0.810	0.642
*No*	MR	157.92	156.22	158.30	159.67	159.85	161.79
*Yes*	MR	163.46	165.41	163.02	161.45	161.24	159.02
Medication errors argument in university courses	U	10,989.00	11,605.00	11,870.00	11,811.00	11,756.00	11,478.00
	Z	−1.417	−0.814	−0.097	−0.219	−0.362	−0.962
	*p*	0.156	0.416	0.923	0.827	0.717	0.336
*No*	MR	165.10	158.95	160.74	161.03	159.70	158.32
*Yes*	MR	152.63	163.15	160.09	159.59	161.87	164.23
Medication errors argument in post university courses	U	12,615.00	12,156.00	12,275.50	12,506.00	12,340.50	12,399.50
	Z	−0.007	−1.172	−0.677	−0.225	−0.606	−0.467
	*p*	0.995	0.241	0.498	0.822	0.545	0.640
*No*	MR	160.47	157.91	158.58	159.87	158.94	161.73
*Yes*	MR	160.53	163.79	162.94	161.30	162.48	158.94
Level of english language knowledge	U	10,105.00	10,685.00	10,407.50	10,375.00	10,412.50	10,547.50
	Z	−0.938	−0.007	−0.599	−0.674	−0.649	−0.323
	*p*	0.348	0.995	0.549	0.500	0.516	0.747
*Insufficient*	MR	163.09	160.49	161.74	159.11	161.72	159.88
*Excellent*	MR	154.37	160.53	157.55	163.79	157.61	161.97
Access to Internet connection	U	4155.00	4681.00	4718.00	3741.00	4708.00	4342.00
	Z	−1.689	−0.737	−0.457	−3.585	−0.539	−1.780
	*p*	0.091	0.416	0.648	<0.001 *	0.590	0.075
*No*	MR	139.71	155.18	156.26	127.53	155.97	175.79
*Yes*	MR	162.97	161.13	161.00	164.42	161.04	158.68
Access to library	U	11,894.00	12,141.00	12,103.50	11,671.00	11,602.50	12,235.50
	Z	−0.544	−0.294	−0.304	−1.177	−1.439	−0.043
	*p*	0.587	0.769	0.761	0.239	0.150	0.966
*No*	MR	159.97	160.59	159.66	159.47	162.25	158.88
*Yes*	MR	161.30	160.36	161.78	162.06	157.85	162.96

U: U di Mann–Whitney; MR: Medium Rank; * statistically significant value. Behav_1: collaboration with the pharmacist. Behav_2: health documentation. Behav_3: support guidelines. Behav_4: medication reconciliation. Behav_5: use of hydroalcoholic gel. Behav_6: vital signs.

**Table 6:** Predictive variable to knowledge, attitude and behavior scores.

	Knowledge Scores			Attitude Scores			Behavior Scores		
Independent Variable	OR (95% CI)	B	*p*	OR (95% CI)	B	*p*	OR (95% CI)	B	*p*
Sex (male vs. female)	0.96 (0.59–1.57)	−0.032	0.897	1.49 (0.868–2.56)	0.401	0.147	0.97 (0.55–1.71)	−0.027	0.925
Title of study (University. vs. Non-uni)	0.43 (0.23–0.77)	−0.842	0.005	0.98 (0.52–1.82)	−0.018	0.954	0.77 (0.42–1.41)	−0.257	0.405
Education post-base (No vs. Yes)	0.90 (0.57–1.40)	−0.104	0.648	0.85 (0.51–1.42)	−0.159	0.545	0.97 (0.58–1.63)	−0.027	0.919
Medication errors argument in university courses (No vs. yes)	0.96 (0.60–1.52)	−0.038	0.870	1.26 (0.74–2.12)	0.233	0.383	0.73 (0.42–1.27)	−0.306	0.274
Medication errors argument in post university courses (No vs. Yes)	1.03 (0.66–1.62)	0.038	0.869	0.84 (0.50–1.42)	−0.164	0.535	0.78 (0.46–1.32)	−0.247	0.357
Access to Internet connection (No vs. Yes)	1.15 (0.56–2.37)	0.146	0.690	0.41 (0.19–0.86)	−0.887	0.018	0.45 (0.21–0.96)	−0.784	0.039
Access to library (No vs. Yes)	1.11 (0.70–1.75)	0.108	0.644	0.80 (0.47–1.37)	−0.212	0.432	0.91 (0.54–1.56)	−0.084	0.755
Level of English language knowledge (insufficient vs. excellent)	1.21 (0.74–1.69)	0.191	0.439	0.79 (0.46–1.37)	−0.226	0.418	1.04 (0.59–1.84)	0.047	0.872
Age (continuous variable)	0.97 (0.95–1.00)	−0.021	0.050	1.01 (0.99–1.03)	0.014	0.247	1.00 (0.97–1.02)	0.001	0.943
Work experience (continuous variable)	0.97 (0.95–1.00)	−0.021	0.054	1.01 (0.99–1.04)	0.013	0.232	1.00 (0.97–1.02)	0.003	0.824

OR: Odds Ratio. B: regression coefficient.

**Table 7:** Regression Model—Behaviors vs. Attitudes and Knowledge.

Independent Variable	N	OR (95% CI)	B	*p*
Attitudes (Negative vs. Positive)				
Inappropriate Behaviors	40 vs. 36	6.02 (3.41–10.62)	1.796	<0.001
Appropriate Behaviors	38 vs. 206			
Knowledge (Inadequate vs. Adequate)				
Inappropriate Behaviors	56 vs. 20	2.33 (1.32–4.12)	0.849	<0.005
Appropriate Behaviors	133 vs. 111			

**Table 8:** Spearman’s rho.

			Knowledge	Attitude	Behavior
Spearman’s Rho	knowledge	Correlation coefficient	1.000		
		Sig. (2-tailed)			
		N	320		
	attitude	Correlation coefficient	0.162 *	1.000	
		Sig. (2-tailed)	0.004	.	
		N	320	320	
	behavior	Correlation coefficient	0.166 *	0.367 *	1.000
		Sig. (2-tailed)	0.003	0.000	.
		*N*	320	320	320

* statistically significant value.

## Data Availability

The data presented in this study are available on request from the corresponding author. The data are not publicly available due to privacy and ethical restrictions.

## Supplementary Materials

The following supporting information can be downloaded at: https://www.mdpi.com/article/10.3390/nursrep16030098/s1, Supplementary file S1: Supplementary Statistical Results for Knowledge, Attitude, and Behaviour Scales.

## Public Involvement Statement

A voluntary and convenience sampling method was used to recruit participants. The survey was conducted via an online questionnaire, advertised on websites dedicated to nursing care and social media platforms.

## Guidelines and Standards Statement

This manuscript was drafted against the STROBE Checklist for cross sectional study.

## Abbreviations

CI	Confidence Interval
LEA	Essential Level of Care
PNRR	National Recovery and Resilience Plan
OR	Odds Ratio
