# Supplementary material for: Knowledge, Attitudes, and Behaviors of Italian Home Care Nurses: Factors Associated with Medication Error Prevention in a Nationwide Cross-Sectional Survey
Source: Nurs Rep. 2026 Mar 14;16(3):98. doi: 10.3390/nursrep16030098 (PMC13028940; doi:10.3390/nursrep16030098)
Supplement: Supplementary file 1 [file nursrep-16-00098-s001.zip › nursrep-4157842-supplementary.pdf]

Supplementary file S1 – Supplementary Statistical Results for Knowledge, Attitude, and Behaviour Scales

Table S1: Descriptive statistics for the knowledge subscale (frequency and percentage)

Item _ Knwoledge Scale	*Strongly disagree*	*Disagree*	*Neutral*	*Agree*	*Strongly agree*	*Inadequate knowledge*	*Adequate knowledge*
Know_1: computerized prescription and administration	13 (4.1)	15 (4.7)	77 (24.1)	106 (33.1)	109 (34.1)	105 (32.8)	215 (67.2)
Know_2: individualized therapy supply	13 (4.1)	20 (6.3)	59 (18.4)	123 (38.4)	105 (32.8)	92 (28.7)	228 (71.3)
Know_3: protocols and informational brochures	6 (1.9)	14 (4.4)	41 (12.8)	132 (41.3)	127 (39.7)	61 (19.1)	259 (80.9)
Know_4: characteristics of the home setting	24 (7.5)	48 (15.00)	83 (25.9)	101 (31.6)	64 (20.00)	155 (48.4)	165 (51.6)
Know_5: workload	4 (1.3)	13 (4.1)	32 (10.00)	97 (30.3)	174 (54.4)	49 (15.3)	174 (51.6)
Know_6: presence of family members and caregivers	10 (3.1)	31 (9.7)	66 (20.6)	101 (31.6)	112 (35.00)	107 (33.4)	213 (66.6)
Know_7: polypharmacy	30 (9.4)	49 (15.3)	49 (15.3)	96 (30.00)	96 (30.00)	128 (40.0)	192 (60.0)

Table S2: Descriptive statistics for the attitude subscale (frequency and percentage)

Item _ Attitude Scale	*Disagree*	*Neutral*	*Agree*	*Negative attitude*	*Positive attitude*
Att_1: continuous education	3 (.9)	49 (15.3)	268 (83.8)	52 (16.3)	268 (83.6)
Att_2: professional awareness	2 (.6)	33 (10.3)	285 (89.1)	35 (10.9)	285 (89.1)
Att_3: professional motivation	8 (2.5)	50 (15.6)	262 (81.9)	58 (18.1)	262 (81.9)
Att_4: protocols, guidelines, and procedures	3 (.9)	48 (15.00)	269 (84.1)	51 (15.9)	269 (84.1)
Att_5: nursing prescription	16 (5.00)	82 (25.6)	222 (69.4)	98 (30.6)	222 (69.4)
Att_6: infusion speed	0 (0)	15 (4.7)	305 (95.3)	15 (4.7)	305 (95.3)
Att_7: the 8G rule	1 (.3)	12 (3.8)	307 (95.9)	13 (4.1)	307 (95.9)

Table S3: Descriptive statistics for the behaviour subscale (frequency and percentage)

Item _ Behaviour Scale	*Strongly disagree*	*Disagree*	*Neutral*	*Agree*	*Strongly agree*	*Incorrect behaviour*	*Correct behaviour*
Behav_1: collaboration with the pharmacist	10 (3.1)	27 (8.4)	72 (22.5)	116 (36.3)	95 (29.7)	109 (34.1)	211 (65.9)
Behav_2: medical documentation	1 (.3)	13 (4.1)	13 (4.1)	146 (45.6)	147 (45.9)	27 (8.4)	293 (91.6)
Behav_3: support guidelines	1 (.3)	24 (7.5)	23 (7.2)	136 (42.5)	136 (42.5)	48 (15.0)	272 (85.0)
Behav_4: pharmacological reconciliation	2 (.6)	22 (6.9)	23 (7.2)	136 (42.5)	137 (42.8)	47 (14.7)	273 (85.3)
Behav_5: use of hydroalcoholic gel	5 (1.6)	7 (2.2)	26 (8.1)	75 (23.4)	207 (64.7)	38 (11.9)	282 (88.1)
Behav_6: vital signs	3 (.9)	5 (1.6)	32 (10.0)	115 (35.9)	165 (51.6)	40 (12.5)	280 (87.5)

Table S4: Results of the Kruskal-Wallis test and post-hoc comparisons performed using the Mann-Whitney test: knowledge section						
Variable		*Variable and categories*	*U*	*Z*	*p*	*Average Rank*
	Age<br>H(1)=1.405; *p*=.236					
		H(1)= 4.166; *p* = .041*				
		0-5 *vs* 6-10 (0,1)	4633.50	-.079	.937	102.82 *vs* 103.36
		0-5 *vs* 11-15 (0,2)	1173.00	-.426	.670	78.44 *vs* 74.67
		0-5 vs 16-20 (0,3)	1224.50	-.061	.952	77.94 vs 78.47
		0-5 *vs* 21-25 (0,4)	1505.00	-2.217	.027*	86.01 *vs* 68.25
		0-5 *vs* 26-30 (0,5)	1534.50	-1.399	.162	83.80 *vs* 72.52
		0-5 *vs* 31-35 (0,6)	1143.50	-1.494	.135	80.65 vs 67.68
		0-5 *vs* 36-40(0,7)	195.50	-.184	.854	70.75 vs 67.17
		0-5 *vs* > 41 (0,8)	107.50	-.665	.506	70.22 vs 55.25
		6-10 *vs* 11-15 (1,2)	579.00	-.445	.656	43.99 vs 41.67
		6-10 *vs* 16-20 (1,3)	611.00	-.014	.989	43.49 vs 43.56
		6-10 *vs* 21-25 (1,4)	742.00	-2.058	.040*	51.59 vs 41.00
		6-10 *vs* 26-30 (1,5)	757.00	-1.329	.184	49.37 vs 42.62
		6-10 *vs* 31-35 (1,6)	564.00	-1.432	.152	46.21 vs 38.70
Know_1		6-10 *vs* 36-40 (1,7)	96.50	-.202	.840	36.08 vs 34.17
		6-10 *vs* >41 (1,8)	53.00	-.676	.499	35.72 vs 28.00
		11-15 *vs* 16-20 (2,3)	153.00	-.357	.721	18.00 vs 19.00
		11-15 *vs* 21-25 (2,4)	210.00	-1.101	.271	25.83 vs 22.00
		11-15 *vs* 26-30 (2,5)	213.00	-.594	.552	23.67 vs 21.69
		11-15 *vs* 31-35 (2,6)	159.00	-.725	.469	20.67 vs 18.45
		11-15 *vs* 36-40 (2,7)	27.00	.000	1.00	11.00 vs 11.00
		11-15 *vs* > 41 (2,8)	15.00	-.457	.648	10.67 vs 9.00
		16-20 *vs* 21-25 (3,4)	196.00	-1.478	.140	26.61 vs 21.50
		16-20 *vs* 26-30 (3,5)	200.00	-.974	.330	24.39 vs 21.19
		16-20 *vs* 31-35 (3,6)	149.00	-1.084	.278	21.22 vs 17.95
		16-20 *vs* 36-40 (3,7)	25.50	-.192	.847	11.08 vs 10.50
		16-20 *vs* >41 (3,8)	14.00	-.634	.526	10.72 vs 8.50
	Work experience	21-25 *vs* 26-30 (4,5)	336.00	-.561	.575	26.50 vs 28.58
		21-25 *vs* 31-35 (4,6)	266.00	-.338	.735	24.00 vs 25.20
		21-25 *vs* 36-40 (4,7)	35.00	-.540	.589	15.75 vs 18.33
		21-25 *vs* > 41 (4,8)	28.00	.000	1.00	15.50 vs 15.50

		26-30 *vs* 31-35 (5,6)	253.00	-.181	.857	23.77 vs 23.15
		26-30 *vs* 36-40 (5,7)	35.50	-.294	.769	14.87 vs 16.17
		26-30 *vs* > 41 (5,8)	24.00	-.208	.835	14.58 vs 13.50
		31-35 *vs* 36-40 (6,7)	26.50	-.372	.710	11.83 vs 13.17
		31-35 *vs* > 41 (6,8)	19.00	-.132	.895	11.55 vs 11.00
		36-40 *vs* > 41 (7,8)	2.50	-.333	.739	3.17 vs 2.75
	Professional training					
		<1 *vs* 1-5 h/week (0-1)	9955.50	-1.179	.239	143.66 *vs* 153.38
		<1 vs 6-10 h/week (0-2)	1176.50	-.097	.922	69.89 vs 70.68
		<1 vs >11 h/week (0-3)	43.00	-1.846	.065	61.64 vs 23.00
		1-5 vs 6-10 h/week (1-2)	1693.00	-.497	.619	100.54 vs 95.18
		1-5 vs >11 h/week (1-3)	53.00	-2.146	.032*	91.70 vs 28.00
		6-10 vs >10 (2-3)	7.00	-1.742	.082	12.15 vs 5.00
	Geographical area					
		North *vs* Center (0,1)	6226.50	-.457	.648	112.24 *vs* 115.54
		North vs South (0,2)	4440.50	-1.490	.136	95.39 vs 105.25
		Center *vs* South (1,2)	5232.50	-1.097	.273	104.24 *vs* 111.74

U: U di Mann-Whitney; H: (Degrees of freedom); * statistically significant value
Know_1: computerized prescription and administration

Table S5: Results of the Kruskal-Wallis test and post-hoc comparisons performed using the Mann-Whitney test: knowledge section

Variable		*Variable and categories*	*U*	*Z*	*p*	*Average Rank*
Know_2	Age<br>H(1)=2.164; *p*=.141					
	Work experience<br>H(1)= .051; *p*= .822					
	Professional training	<1 *vs* 1-5 h/week (0-1)	10552.50	-.171	.864	148.68 *vs* 150.05
		<1 vs 6-10 h/week (0-2)	1137.50	-.402	.688	69.56 vs 72.63
		<1 *vs* >11 h/week (0-3)	94.50	-.629	.529	61.21 *vs* 48.75
		1-5 vs 6-10 h/week (1-2)	1727.50	-.329	.743	99.56 vs 103.13
		1-5 vs >11 h/week (1-3)	140.50	-.667	.505	91.22 vs 71.75
		6-10 vs >10 (2-3)	15.00	-.740	.460	11.75 vs 9.00
	Geographical area					
		North *vs* Center (0,1)	5578.50	-2.159	.031*	121.87 *vs* 107.10
		North vs South (0,2)	4567.50	-1.187	.235	103.41 vs 96.11
		Center vs South (1,2)	5307.00	-.879	.379	104.86 vs 110.94

U: U di Mann-Whitney; H: (Degrees of freedom); * statistically significant value
Know_2: individualized therapy provision

Table S6: Results of the Kruskal-Wallis test and post-hoc comparisons performed using the Mann-Whitney test: knowledge section

Variable		*Variable and categories*	*U*	*Z*	*p*	*Average Rank*
Know_3	Age<br>H(1)=1.995; *p*=.158					
	Work experience<br>H(1)= .411; *p*= .521					
	Professional training	<1 *vs* 1-5 h/week (0-1)	10049	-1.229	.219	144.45 *vs* 152.86
		<1 *vs* 6-10 h/week (0-2)	1093.0	-.798	.425	70.82 *vs* 65.15
		<1 *vs* >11 h/week (0-3)	93.00	-.743	.458	60.78 *vs* 74.00
		1-5 *vs* 6-10 h/week (1-2)	1543.0	-1.533	.125	101.38 *vs* 87.65
		1-5 *vs* >11 h/week (1-3)	150.00	-.619	.536	90.84 *vs* 105.50
		6-10 *vs* >10 (2-3)	14.00	-.887	.375	11.20 *vs* 14.50
	Geographical area					
		North *vs* Center (0,1)	6192.00	-.638	.524	116.08 *vs* 112.17
		North *vs* South (0,2)	4741.00	-.711	.477	98.23 *vs* 102.02
		Center *vs* South (1,2)	5218.00	-1.335	.182	104.12 *vs* 111.89

U: U di Mann-Whitney; H: (Degrees of freedom); * statistically significant value
Know_3: protocols and information brochures

Table S7: Results of the Kruskal-Wallis test and post-hoc comparisons performed using the Mann-Whitney test: knowledge section

Variable		*Variable and categories*	*U*	*Z*	*p*	*Average Rank*
Know_4	Age<br>H(1)=2.493; *p*=.114					
	Work experience<br>H(1)= 3.094; *p*= .079					
	Professional training	<1 *vs* 1-5 h/week (0-1)	10605.50	-.071	.943	149.88 *vs* 149.25
		<1 vs 6-10 h/week (0-2)	1026.50	-1.135	.256	68.63 vs 78.18
		<1 vs >11 h/week (0-3)	58.00	-1.432	.152	61.51 vs 30.50
		1-5 vs 6-10 h/week (1-2)	1536.50	-1.200	.230	98.58 vs 112.68
		1-5 vs >11 h/week (1-3)	88.00	-1.426	.154	91.51 vs 45.50
		6-10 vs >10 (2-3)	7.00	-1.742	.082	12.15 vs 10.00
	Geographical area					
		North *vs* Center (0,1)	6016.00	-.931	.352	117.75 *vs* 110.72
		North *vs* South (0,2)	4471.00	-1.306	.192	104.32 *vs* 95.08
		Center *vs* South (1,2)	5452.00	-.449	.654	108.94 *vs* 105.62

U: U di Mann-Whitney; H: (Degrees of freedom); * statistically significant value
Know_4: characteristics of the home setting

**Table S8**: Results of the Kruskal-Wallis test and post-hoc comparisons performed using the Mann-Whitney test: knowledge section						
Variable		*Variable and categories*	*U*	*Z*	*p*	*Average Rank*
Know_5	H(1)=4.853; *p*=.028*					
	Age	20-25 *vs* 26-30 (0-1)	1692.00	-.013	.990	64.47 *vs* 64.52
		20-25 vs 31-35 (0-2)	1446.00	-.082	.935	54.65 vs 54.37
		20-25 *vs* 36-40 (0-3)	436.50	-.527	.598	35.44 *vs* 33.75
		20-25 *vs* 41-45 (0,4)	696.00	-.320	.749	40.35 *vs* 39.36
		20-25 *vs* 46-50 (0,5)	856.50	-1.697	.090	48.21 *vs* 41.96
		20-25 vs 51-55 (0,6)	867.00	.000	1.00	43.00 vs 43.00
		20-25 *vs* 56-60 (0,7)	243.00	-2.260	.024*	34.24 *vs* 25.69
		20-25 *vs* >61 (0,8)	60.00	-1.071	.284	27.82 *vs* 22.00
		26-30 *vs* 31-35 (1,2)	2181.50	-.104	.917	67.67 *vs* 67.27
		26-30 *vs* 36-40 (1,3)	658.50	-.569	.569	48.45 *vs* 46.08
		26-30 *vs* 41-45(1,4)	1050.00	-.356	.722	53.36 *vs* 52.00
		26-30 *vs* 46-50 (1,5)	1292.00	-1.910	.056	61.22 *vs* 53.13
		26-30 *vs* 51-55 (1,6)	1308.00	-.011	.991	56.01 *vs* 55.97
		26-30 *vs* 56-60 (1,7)	366.50	-2.450	.014*	47.24 *vs* 35.19
		26-30 *vs* >61 (1,8)	90.50	-1.105	.269	40.82 *vs* 32.17
		31-35 *vs* 36-40 (2,3)	490.50	-.474	.635	38.39 *vs* 36.75
		31-35 *vs* 41-45 (2,4)	782.00	-.257	.797	43.28 *vs* 42.43
		31-35 *vs* 46-50 (2,5)	963.00	-1.675	.094	51.11 *vs* 44.69
		31-35 *vs* 51-55 (2,6)	964.00	-.703	.942	45.91 *vs* 46.15
		31-35 *vs* 56-60 (2,7)	273.50	-2.244	.025*	37.20 *vs* 28.04
		31-35 *vs* >61 (2,8)	67.50	-1.037	.300	30.82 *vs* 24.50
		36-40 *vs* 41-45 (3,4)	246.00	-.217	.828	23.17 *vs* 23.17
		36-40 *vs* 45-50 (3,5)	319.50	-.744	.457	30.75 *vs* 28.19
		36-40 *vs* 51-55 (3,6)	291.00	-.488	.626	25.67 *vs* 26.94
		36-40 *vs* 56-60 (3,7)	91.50	-1.346	.178	17.42 *vs* 14.04
		36-40 *vs* >61 (3,8)	22.50	-.664	.507	11.25 *vs* 9.50
		41-45 *vs* 46-50 (4,5)	484.00	-1.119	.263	36.21 *vs* 32.41
		41-45 *vs* 51-55 (4,6)	464.00	-.292	.770	31.07 *vs* 31.85
		41-45 *vs* 56-60 (4,7)	138.00	-1.719	.086	22.57 *vs* 17.62
		41-45 *vs* >61 (4,8)	34.00	-.839	.402	16.29 *vs* 13.33

	46-50 *vs* 51-55 (5,6)	571.00	-1.492	.136	36.64 *vs* 39.71
	46-50 *vs* 56-60 (5,7)	221.00	-.875	.382	27.33 *vs* 24.00
	46-50 *vs* >61 (5,8)	54.00	-.289	.773	21.62 *vs* 20.00
	51-55 *vs* 56-60 (6,7)	162.00	-2.058	.040*	25.74 *vs* 19.46
	51-55 *vs* >61 (6,8)	40.00	-1.033	.301	19.32 *vs* 15.33
	56-60 *vs* >61 (7,8)	18.50	-.160	.873	8.42 *vs* 8.83
H(1)=3.972; *p*=.046*					
Work experience	0-5 *vs* 6-10 (0,1)	4628.00	-.132	.895	102.78 *vs* 103.44
	0-5 vs 11-15 (0,2)	1180.50	-.505	.614	78.38 vs 75.08
	0-5 *vs* 16-20 (0,3)	1080.00	-1.579	.114	76.88 *vs* 86.50
	0-5 *vs* 21-25 (0,4)	1745.00	-1.252	.211	84.26 *vs* 76.82
	0-5 *vs* 26-30 (0,5)	1522.50	-1.909	.056	83.89 *vs* 72.06
	0-5 *vs* 31-35 (0,6)	1197.50	-1.511	.131	80.26 *vs* 70.38
	0-5 *vs* 36-40 (0,7)	25.50	-4.273	<.001*	71.81 *vs* 10.50
	0-5 *vs* > 41 (0,8)	120.00	-.530	.596	69.88 *vs* 78.50
	6-10 *vs* 11-15 (1,2)	582.00	-.550	.582	43.94 *vs* 41.83
	6-10 *vs* 16-20 (1,3)	540.00	-1.519	.129	42.44 *vs* 47.50
	6-10 *vs* 21-25 (1,4)	860.00	-1.213	.225	49.85 *vs* 45.21
	6-10 *vs* 26-30 (1,5)	750.00	-1.786	.074	49.47 *vs* 42.35
	6-10 *vs* 31-35 (1,6)	590.00	-1.458	.145	45.82 *vs* 40.00
	6-10 *vs* 36-40 (1,7)	12.00	-4.104	<.001*	37.32 *vs* 6.00
	6-10 *vs* >41 (1,8)	60.00	-.512	.609	35.38 *vs* 39.50
	11-15 *vs* 16-20 (2,3)	135.00	-1.784	.074	17.00 *vs* 20.00
	11-15 *vs* 21-25 (2,4)	240.00	-.393	.694	24.17 *vs* 23.07
	11-15 *vs* 26-30 (2,5)	210.00	-.789	.430	23.83 *vs* 21.58
	11-15 *vs* 31-35 (2,6)	165.00	-.621	.535	20.33 *vs* 18.75
	11-15 *vs* 36-40 (2,7)	4.50	-2.887	.004*	12.25 *vs* 3.50
	11-15 *vs* > 41 (2,8)	15.00	-.610	.542	10.33 *vs* 12.00
	16-20 *vs* 21-25 (3,4)	198.00	-2.083	.037*	26.50 *vs* 21.57
	16-20 *vs* 26-30 (3,5)	171.00	-2.373	.018*	26.00 *vs* 20.08
	16-20 *vs* 31-35 (3,6)	135.00	-2.246	.025*	22.00 *vs* 17.25
	16-20 *vs* 36-40 (3,7)	.000	-4.472	<.001*	12.50 *vs* 2.00
	16-20 *vs* >41 (3,8)	18.00	.000	1.00	10.50 *vs* 10.50
	21-25 *vs* 26-30 (4,5)	344.00	-.467	.640	28.21 *vs* 26.73
	21-25 *vs* 31-35 (4,6)	270.00	-.287	.774	24.86 *vs* 24.00
	21-25 *vs* 36-40 (4,7)	9.00	-2.803	.005*	17.18 *vs* 5.00
	21-25 *vs* > 41 (4,8)	22.00	-.720	.472	15.29 *vs* 18.50

	26-30 *vs* 31-35 (5,6)	255.00	-.146	.884	23.31 *vs* 23.75
	26-30 *vs* 36-40 (5,7)	10.50	-2.478	.013*	16.10 *vs* 5.50
	26-30 *vs* > 41 (5,8)	19.00	-.832	.405	14.23 *vs* 18.00
	31-35 *vs* 36-40 (6,7)	7.50	-2.487	.013*	13.13 *vs* 4.50
	31-35 *vs* > 41 (6,8)	15.00	-.786	.432	11.25 *vs* 28.00
	36-40 *vs* > 41 (7,8)	.000	-2.00	.046*	2.00 *vs* 4.50
Professional training					
	<1 vs 1-5 h/week (0-1)	10437.50	-.476	.634	147.71 *vs* 150.69
	<1 vs 6-10 h/week (0-2)	1082.50	-.985	.324	70.90 *vs* 64.63
	<1 vs >11 h/week (0-3)	100.00	-.613	.540	60.84 *vs* 70.50
	1-5 vs 6-10 h/week (1-2)	1592.50	-1.305	.192	101.10 *vs* 90.13
	1-5 vs >11 h/week (1-3)	154.00	-.568	.570	90.86 *vs* 103.50
	6-10 vs >11 h/week (2-3)	15.00	-.786	.432	11.25 *vs* 14.00
Geographical area					
	North *vs* Center (0,1)	6412.00	-.003	.997	114.01 *vs* 113.99
	North *vs* South (0,2)	4573.00	-1.363	.173	103.36 *vs* 96.17
	Center *vs* South (1,2)	5221.00	-1.410	.159	110.85 *vs* 103.14

U: U di Mann-Whitney; H: (Degrees of freedom); * statistically significant value
Know_5: workload

Table S9: Results of the Kruskal-Wallis test and post-hoc comparisons performed using the Mann-Whitney test: knowledge section

Variable		*Variable and categories*	*U*	*Z*	*p*	*Average Rank*
Know_6	H(1)=15.433; *p*= < .001*					
	Age	20-25 *vs* 26-30 (0-1)	1762.00	-1.294	.196	68.45 *vs* 61.88
		20-25 *vs* 31-35 (0-2)	1381.50	-.651	.538	55.91 *vs* 53.24
		20-25 *vs* 36-40 (0-3)	396.00	-1.178	.239	36.24 *vs* 31.50
		20-25 *vs* 41-45 (0,4)	548.00	-2.191	.028*	43.25 *vs* 34.07
		20-25 *vs* 46-50 (0,5)	781.50	-2.209	.027*	49.68 *vs* 40.04
		20-25 *vs* 51-55 (0,6)	578.00	-3.184	.001*	48.67 *vs* 34.50
		20-25 *vs* 56-60 (0,7)	243.50	-1.958	.050	34.23 *vs* 25.73
		20-25 *vs* >61 (0,8)	40.50	-1.888	.059	28.21 *vs* 15.50
		26-30 *vs* 31-35 (1,2)	2078.00	-.677	.498	65.99 *vs* 69.54
		26-30 *vs* 36-40 (1,3)	669.00	-.286	.775	48.31 *vs* 46.67
		26-30 *vs* 41-45(1,4)	938.00	-1.242	.214	54.82 *vs* 48.00
		26-30 *vs* 46-50 (1,5)	1334.00	-1.196	.232	60.68 *vs* 54.21

	26-30 *vs* 51-55 (1,6)	1007.00	-2.311	.021*	59.92 *vs* 47.12
	26-30 *vs* 56-60 (1,7)	419.00	-1.156	.248	46.56 *vs* 39.23
	26-30 *vs* >61 (1,8)	73.00	-1.341	.180	41.05 *vs* 26.33
	31-35 *vs* 36-40 (2,3)	468.00	-.729	.466	38.79 *vs* 35.50
	31-35 *vs* 41-45 (2,4)	652.00	-1.710	.087	45.56 *vs* 37.79
	31-35 *vs* 46-50 (2,5)	928.50	-1.700	.089	51.71 *vs* 43.81
	31-35 *vs* 51-55 (2,6)	694.00	-2.728	.006*	50.82 *vs* 37.91
	31-35 *vs* 56-60 (2,7)	290.50	-1.544	.123	36.90 *vs* 29.35
	31-35 *vs* >61 (2,8)	49.50	-1.594	.111	31.13 *vs* 18.50
	36-40 *vs* 41-45 (3,4)	228.00	-.639	.523	24.83 *vs* 22.64
	36-40 *vs* 46-50 (3,5)	324.00	-.550	.583	30.50 *vs* 28.31
	36-40 *vs* 51-55 (3,6)	246.00	-1.336	.181	29.83 *vs* 24.74
	36-40 *vs* 56-60 (3,7)	102.00	-.711	.477	16.83 *vs* 14.85
	36-40 *vs* >61 (3,8)	18.00	-1.074	.283	11.50 *vs* 8.00
	41-45 *vs* 46-50 (4,5)	536.00	-.149	.882	33.64 *vs* 34.26
	41-45 *vs* 51-55 (4,6)	428.00	-.784	.433	33.21 *vs* 30.09
	41-45 *vs* 56-60 (4,7)	176.00	-.196	.845	21.21 *vs* 20.54
	41-45 *vs* >61 (4,8)	32.00	-.775	.438	16.36 *vs* 12.67
	46-50 *vs* 51-55 (5,6)	584.00	-1.011	.312	39.03 *vs* 34.68
	46-50 *vs* 56-60 (5,7)	240.50	-.321	.748	26.83 *vs* 25.50
	46-50 *vs* >61 (5,8)	43.50	-.854	.393	21.88 *vs* 16.50
	51-55 *vs* 56-60 (6,7)	206.00	-.412	.680	23.56 *vs* 25.15
	51-55 *vs* >61 (6,8)	44.00	-.451	.652	19.21 *vs* 16.67
	56-60 *vs* >61 (7,8)	15.50	-.620	.535	8.81 *vs* 7.17
H(1)=10.229; *p*=.001*					
Work experience	0-5 *vs* 6-10 (0,1)	4170.00	-1.564	.118	106.56 *vs* 95.82
	0-5 *vs* 11-15 (0,2)	1196.50	-.271	.786	78.27 *vs* 75.97
	0-5 *vs* 16-20 (0,3)	922.50	-2.236	.025*	80.27 *vs* 60.75
	0-5 *vs* 21-25 (0,4)	1572.00	-1.934	.053	85.53 *vs* 70.64
	0-5 *vs* 26-30 (0,5)	1401.00	-2.209	.027*	84.77 *vs* 67.38
	0-5 *vs* 31-35 (0,6)	1162.00	-1.428	.153	80.52 *vs* 68.60
	0-5 *vs* 36-40 (0,7)	51.00	-2.911	.004*	71.63 *vs* 19.00
	0-5 *vs* > 41 (0,8)	103.00	-.808	.419	69.75 *vs* 87.00
	6-10 *vs* 11-15 (1,2)	566.00	-.596	.551	42.82 *vs* 46.06
	6-10 *vs* 16-20 (1,3)	522.00	-1.134	.257	44.82 *vs* 38.50

	6-10 *vs* 21-25 (1,4)	880.00	-.692	.489	49.56 *vs* 45.93
	6-10 *vs* 26-30 (1,5)	788.00	-.946	.335	48.91 *vs* 43.81
	6-10 *vs* 31-35 (1,6)	648.00	-.382	.702	44.97 *vs* 42.90
	6-10 *vs* 36-40 (1,7)	36.00	-2.243	.025*	36.97 *vs* 14.00
	6-10 *vs* >41 (1,8)	44.00	-1.029	.303	31.15 *vs* 47.50
	11-15 *vs* 16-20 (2,3)	126.00	-1.348	.178	20.50 *vs* 16.50
	11-15 *vs* 21-25 (2,4)	214.00	-1.023	.306	25.61 *vs* 22.14
	11-15 *vs* 26-30 (2,5)	191.00	-1.217	.224	24.89 *vs* 20.85
	11-15 *vs* 31-35 (2,6)	158.00	-.728	.434	20.72 *vs* 18.40
	11-15 *vs* 36-40 (2,7)	7.50	-2.327	.020*	12.08 *vs* 4.50
	11-15 *vs* > 41 (2,8)	13.00	-.839	.402	10.22 *vs* 13.00
	16-20 *vs* 21-25 (3,4)	234.00	-.469	.639	22.50 *vs* 24.14
	16-20 *vs* 26-30 (3,5)	225.00	-.248	.804	22.00 *vs* 22.85
	16-20 *vs* 31-35 (3,6)	162.00	-.611	.541	18.50 *vs* 20.40
	16-20 *vs* 36-40 (3,7)	13.50	-1.581	.114	11.75 *vs* 6.50
	16-20 *vs* >41 (3,8)	9.00	-1.314	.189	10.00 *vs* 15.00
	21-25 *vs* 26-30 (4,5)	352.00	-.241	.809	27.93 *vs* 27.04
	21-25 *vs* 31-35 (4,6)	272.00	-.196	.845	24.21 *vs* 24.90
	21-25 *vs* 36-40 (4,7)	18.00	-1.852	.064	16.86 *vs* 8.00
	21-25 *vs* > 41 (4,8)	16.00	-1.175	.240	15.07 *vs* 21.50
	26-30 *vs* 31-35 (5,6)	244.00	-.413	.680	22.88 *vs* 24.30
	26-30 *vs* 36-40 (5,7)	18.00	-1.736	.082	15.81 *vs* 8.00
	26-30 *vs* > 41 (5,8)	14.00	-1.248	.212	14.04 *vs* 20.50
	31-35 *vs* 36-40 (6,7)	12.00	-1.897	.058	12.90 *vs* 6.00
	31-35 *vs* > 41 (6,8)	12.00	-1.095	.273	11.10 *vs* 15.50
	36-40 *vs* > 41 (7,8)	.000	-2.00	.046*	2.00 *vs* 4.50
Professional training					
	<1 *vs* 1-5 h/week (0-1)	10273.0	-.635	.525	152.67 *vs* 147.39
	<1 *vs* 6-10 h/week (0-2)	1084.00	-.785	.433	70.89 *vs* 64.70
	<1 *vs* >11 h/week (0-3)	82.00	-.943	.346	60.69 *vs* 79.50
	1-5 *vs* 6-10 h/week (1-2)	1694.00	-.475	.635	100.54 *vs* 95.20
	1-5 *vs* >11 h/week (1-3)	117.00	-1.024	.306	90.65 *vs* 122.00
	6-10 *vs* >11 h/week (2-3)	12.00	-1.095	.273	11.10 *vs* 15.50
Geographical area					
	North *vs* Center (0,1)	6128.00	-.706	.480	111.31 *vs* 116.36
	North *vs* South (0,2)	4805.00	-.371	.711	98.83 *vs* 101.33

		Center *vs* South (1,2)	5518.00	-.298	.765	108.40 *vs* 106.33
U: U di Mann-Whitney; H: (Degrees of freedom); * statistically significant value<br>Know_6: presence of family members and caregivers						

Table S10: Results of the Kruskal-Wallis test and post-hoc comparisons performed using the Mann-Whitney test: knowledge section

Variable		*Variable and categories*	*U*	*Z*	*p*	*Average Rank*
Know_7	Age<br>H(1)=2.057; *p*=.152					
	Work experience<br>H(1)= 1.677; *p*= .195					
	Professional training	<1 *vs* 1-5 h/week (0-1)	10072.00	-.931	.352	144.64 *vs* 152.73
		<1 *vs* 6-10 h/week (0-2)	957.50	-1.634	.102	68.05 *vs* 81.63
		<1 *vs* >11 h/week (0-3)	66.00	-1.254	.210	60.55 *vs* 87.50
		1-5 *vs* 6-10 h/week (1-2)	1537.50	-1.232	.218	98.59 *vs* 112.63
		1-5 *vs* >11 h/week (1-3)	109.00	-1.126	.260	90.61 *vs* 126.00
		6-10 *vs* >10 (2-3)	15.00	-.786	.432	11.25 *vs* 14.00
	Geographical area					
		North *vs* Center (0,1)	5600.50	-1.959	.050	121.67 *vs* 107.29
		North *vs* South (0,2)	4237.50	-2.032	.042*	106.52 *vs* 92.56
		Center *vs* South (1,2)	5550.00	-.198	.843	108.13 *vs* 106.68
U: U di Mann-Whitney; H: (Degrees of freedom); * statistically significant value<br>Know_7: polytherapy						

Table S11: Results of the Kruskal-Wallis test and post-hoc comparisons performed using the Mann-Whitney test: attitude section

Variable	*Variable and categories*		*U*	*Z*	*p*	*Average Rank*
Att_1	Age<br>H(1)=1.057; *p*=.300					
	Work experience<br>H(1)= .956; *p*= .328					
	Professional training	<1 *vs* 1-5 h/week (0-1)	10288.00	-.775	.438	146.45 *vs* 151.53
		<1 vs 6-10 h/week (0-2)	1148.50	-.374	.708	69.65 vs 72.08
		<1 vs >11 h/week (0-3)	97.00	-.669	.503	60.82 vs 72.00
		1-5 vs 6-10 h/week (1-2)	1788.50	-.010	.992	99.99 vs 100.08
		1-5 vs >11 h/week (1-3)	152.00	-.594	.553	90.85 vs 104.50
		6-10 vs >10 (2-3)	17.00	-.576	.565	11.65 vs 13.00

	Geographical area					
		North *vs* Center (0,1)	5579.50	-2.699	.007*	121.86 *vs* 107.11
		North *vs* South (0,2)	4446.50	-2.039	.041*	104.55 *vs* 94.81
		Center *vs* South (1,2)	5446.00	-.579	.562	106.01 *vs* 109.44

U: U di Mann-Whitney; H: (Degrees of freedom); * statistically significant value
Att_1: continuing education

Table S12: Results of the Kruskal-Wallis test and post-hoc comparisons performed using the Mann-Whitney test: attitude section

Variable		*Variable and categories*	*U*	*Z*	*p*	*Average Rank*
Att_2	Age<br>H(1)=3.363; *p*=.067					
	Work experience<br>H(1)= 3.067; *p*= .080					
	Professional training	<1 *vs* 1-5 h/week (0-1)	9902.50	-1.970	.049*	143.21 *vs* 153.68
		<1 vs 6-10 h/week (0-2)	1062.50	-1.210	.226	71.07 vs 63.63
		<1 vs >11 h/week (0-3)	102.00	-.574	.566	60.86 vs 69.50
		1-5 vs 6-10 h/week (1-2)	1472.50	-2.616	.009*	101.77 vs 84.13
		1-5 vs >11 h/week (1-3)	166.00	-.394	.693	90.93 vs 97.50
		6-10 vs >10 (2-3)	15.00	-.786	.432	11.25 vs 14.00
	Geographical area					
		North *vs* Center (0,1)	6389.50	-.089	.929	114.22 *vs* 113.81
		North *vs* South (0,2)	4857.50	-.325	.745	100.67 *vs* 99.23
		Center *vs* South (1,2)	5565.50	-.249	.804	108.00 *vs* 106.84

U: U di Mann-Whitney; H: (Degrees of freedom); * statistically significant value
Att_2: nurses' professional awareness

Table S13: Results of the Kruskal-Wallis test and post-hoc comparisons performed using the Mann-Whitney test: attitude section

Variable		*Variable and categories*	*U*	*Z*	*p*	*Average Rank*
Att_3	Age<br>H(1)=1.233; p=.267					
	Work experience<br>H(1)= 1.306; p= .253					
		<1 *vs* 1-5 h/week (0-1)	9930.00	-1.493	.135	143.45 *vs* 153.53
		<1 *vs* 6-10 h/week (0-2)	1152.50	-.312	.755	70.32 *vs* 68.13
		<1 *vs* >11 h/week (0-3)	93.00	-.743	.458	60.78 *vs* 74.00
		1-5 vs 6-10 h/week (1-2)	1612.50	-1.142	.253	100.99 vs 91.13
		1-5 vs >11 h/week (1-3)	152.00	-.594	.553	90.85 vs 104.50
		6-10 vs >10 (2-3)	15.00	-.786	.432	11.25 vs 14.00
	G eo gr					

		North vs Center (0,1)	6283.00	-.395	.693	115.23 vs 112.93
		North vs South (0,2)	4865.00	-.239	.811	100.60 vs 99.31
		Center vs South (1,2)	5585.50	-.135	.892	107.16 vs 107.94

U: U di Mann-Whitney; H: (Degrees of freedom); * statistically significant value
Att_3: nurses' motivation

Table S14: Results of the Kruskal-Wallis test and post-hoc comparisons performed using the Mann-Whitney test: attitude section

Variable		*Variable and categories*	*U*	*Z*	*p*	*Average Rank*
Att_4	Age<br>H(1)=1.873; *p*=.171					
	Work experience<br>H(1)= 3.205; *p*= .073					
	Professional training	<1 *vs* 1-5 h/week (0-1)	10267.50	-.856	.392	152.72 *vs* 147.36
		<1 *vs* 6-10 h/week (0-2)	983.00	-2.003	.045*	71.74 *vs* 59.65
		<1 *vs* >11 h/week (0-3)	74.50	-1.542	.123	61.37 *vs* 38.75
		1-5 *vs* 6-10 h/week (1-2)	1543.00	-1.533	.125	101.38 *vs* 87.65
		1-5 *vs* >11 h/week (1-3)	118.50	-1.275	.202	91.34 *vs* 60.75
		6-10 *vs* >10 (2-3)	16.00	-.566	.572	11.70 *vs* 9.50
	Geographical area					
		North *vs* Center (0,1)	6366.50	-.154	.877	114.44 *vs* 113.62
		North *vs* South (0,2)	4672.50	-.982	.326	102.42 *vs* 97.24
		Center *vs* South (1,2)	5374.50	-.866	.386	109.58 *vs* 104.79

U: U di Mann-Whitney; H: (Degrees of freedom); * statistically significant value
Att_4: protocols, guidelines, and procedures

Table S15: Results of the Kruskal-Wallis test and post-hoc comparisons performed using the Mann-Whitney test: attitude section

Variable		*Variable and categories*	*U*	*Z*	*p*	*Average Rank*
Att_5	Age<br>H(1)=1.656; *p*=.198					
	Work experience<br>H(1)= 2.238; *p*= .135					
	Professional training	<1 *vs* 1-5 h/week (0-1)	10283.50	-.626	.531	146.42 *vs* 151.55
		<1 vs 6-10 h/week (0-2)	1028.00	-1.207	.228	68.64 vs 78.10
		<1 vs >11 h/week (0-3)	79.00	-.998	.318	60.66 vs 81.00
		1-5 vs 6-10 h/week (1-2)	1608.00	-.947	.344	98.98 vs 109.10
		1-5 vs >11 h/week (1-3)	125.00	-.925	.355	90.70 vs 118.00
		6-10 vs >10 (2-3)	16.00	-.683	.495	11.30 vs 13.50

	Geographical area					
		North *vs* Center (0,1)	5714.50	-1.762	.078	120.59 *vs* 108.23
		North vs South (0,2)	4753.50	-.562	.574	101.66 vs 98.11
		Center vs South (1,2)	5214.00	-1.126	.260	104.09 vs 11.94

U: U di Mann-Whitney; H: (Degrees of freedom); * statistically significant value
Att_5: nurse prescribing

Table S16: Results of the Kruskal-Wallis test and post-hoc comparisons performed using the Mann-Whitney test: attitude section

Variable		*Variable and categories*	*U*	*Z*	*p*	*Average Rank*
Att_6	Age<br>H(1)=.155; *p*=.694					
	Work experience<br>H(1)= .130; *p*= .718					
	Professional training	<1 *vs* 1-5 h/week (0-1)	10530.00	-.468	.640	148.49 *vs* 150.17
		<1 vs 6-10 h/week (0-2)	1131.00	-.878	.380	70.50 vs 67.05
		<1 vs >11 h/week (0-3)	113.00	-.324	.746	60.95 vs 64.00
		1-5 vs 6-10 h/week (1-2)	1681.00	-1.240	.215	100.61 vs 94.55
		1-5 vs >11 h/week (1-3)	172.00	-.284	.776	90.96 vs 94.50
		6-10 vs >10 (2-3)	18.00	-.458	.647	11.40 vs 12.50
	Geographical area					
		North *vs* Center (0,1)	6254.50	-.828	.407	112.50 *vs* 115.31
		North vs South (0,2)	4762.50	-1.086	.278	98.43 vs 101.79
		Center vs South (1,2)	5575.50	-.346	.730	107.08 vs 108.05

U: U di Mann-Whitney; H: (Degrees of freedom); * statistically significant value
Att_6: infusion rate

Table S17: Results of the Kruskal-Wallis test and post-hoc comparisons performed using the Mann-Whitney test: attitude section

Variable		*Variable and categories*	*U*	*Z*	*p*	*Average Rank*
Att_7	Age<br>H(1)=1.569; *p*=.210					
	Work experience<br>H(1)= .2.448; *p*= .118					
	Professional training	<1 *vs* 1-5 h/week (0-1)	10321.50	-1.326	.185	146.74 *vs* 151.34
		<1 vs 6-10 h/week (0-2)	1179.50	-.156	.876	69.91 vs 70.53
		<1 vs >11 h/week (0-3)	112.00	-.352	.725	60.95 vs 64.50
		1-5 vs 6-10 h/week (1-2)	1750.50	-.546	.585	100.22 vs 98.03
		1-5 vs >11 h/week (1-3)	174.00	-.239	.811	90.97 vs 93.50

		6-10 vs >10 (2-3)	19.00	-.316	.752	11.45 vs 12.00
	Geographical area					
		North *vs* Center (0,1)	6344.50	-.358	.720	114.65 *vs* 113.43
		North vs South (0,2)	4749.50	-1.495	.135	98.31vs 101.93
		Center vs South (1,2)	5361.50	-1.796	.072	105.31 vs 110.35

U: U di Mann-Whitney; H: (Degrees of freedom); * statistically significant value
Att_7**:** the "8 rights" rule

Table S18: Results of the Kruskal-Wallis test and post-hoc comparisons performed using the Mann-Whitney test: behaviour section

Variable		*Variable and categories*	*U*	*Z*	*p*	*Average Rank*
	H(1)=12.831; *p*= < .001*					
		20-25 *vs* 26-30 (0-1)	1650.50	-1.767	.077	58.36 *vs* 68.56
		20-25 vs 31-35 (0-2)	1012.50	-3.214	.001*	45.85 vs 62.24
		20-25 vs 36-40 (0-3)	334.50	-1.966	.049*	32.56 vs 41.92
		20-25 vs 41-45 (0,4)	577.00	-1.622	.105	37.31 vs 44.89
		20-25 vs 46-50 (0,5)	729.00	-2.519	.012*	40.29 vs 52.31
		20-25 vs 51-55 (0,6)	569.50	-3.130	.002*	37.17 vs 51.57
		20-25 vs 56-60 (0,7)	226.00	-2.034	.042*	30.43 vs 40.62
		20-25 vs >61 (0,8)	60.00	-.721	.471	27.18 vs 33.00
		26-30 vs 31-35 (1,2)	1878.50	-1.748	.080	63.40 vs 73.04
		26-30 vs 36-40 (1,3)	615.50	-.881	.378	46.99 vs 52.31
		26-30 vs 41-45(1,4)	1043.00	-.302	.763	52.55 vs 54.25
Behav_1		26-30 vs 46-50 (1,5)	1340.00	-1.140	.254	56.40 vs 62.64
		26-30 vs 51-55 (1,6)	1068.50	-1.884	.060	52.88 vs 63.07
		26-30 vs 56-60 (1,7)	421.00	-1.903	.274	44.47 vs 51.62
		26-30 vs >61 (1,8)	109.00	-.195	.845	40.42 vs 42.67
		31-35 vs 36-40 (2,3)	496.50	-.272	.786	38.29 vs 37.08
		31-35 vs 41-45 (2,4)	709.00	-1.067	.286	44.56 vs 39.82
		31-35 vs 46-50 (2,5)	1071.00	-.397	.691	49.21 vs 47.46
		31-35 vs 51-55 (2,6)	930.50	-.433	.665	45.32 vs 47.13
		31-35 vs 56-60 (2,7)	365.00	-.112	.911	35.40 vs 35.92
		31-35 vs >61 (2,8)	78.00	-.339	.735	30.63 vs 28.00
		36-40 vs 41-45 (3,4)	232.00	-.554	.579	24.61 vs 22.79
		36-40 vs 46-50 (3,5)	349.50	-.033	.974	29.08 vs 28.96
	Age	36-40 vs 51-55 (3,6)	284.00	-.580	.562	25.28 vs 27.15

	36-40 vs 56-60 (3,7)	111.50	-.290	.772	15.96 vs 16.42
	36-40 vs >61 (3,8)	25.50	-.192	.847	11.08 vs 10.50
	41-45 vs 46-50 (4,5)	505.00	-.847	.397	34.50 vs 33.64
	41-45 vs 51-55 (4,6)	404.00	-1.318	.188	28.93 vs 33.62
	41-45 vs 56-60 (4,7)	159.00	-.799	.424	20.18 vs 22.77
	41-45 vs >61 (4,8)	41.00	-.081	.936	15.96 vs 16.33
	46-50 vs 51-55 (5,6)	612.50	-.748	.454	35.71 vs 38.49
	46-50 vs 56-60 (5,7)	240.50	-.358	.721	26.17 vs 27.50
	46-50 vs >61 (5,8)	55.50	-.187	.852	21.58 vs 20.50
	51-55 vs 56-60 (6,7)	215.50	-.184	.854	24.16 vs 23.58
	51-55 vs >61 (6,8)	44.50	-.507	.612	19.19 vs 16.83
	56-60 vs >61 (7,8)	17.50	-.358	.720	8.65 vs 7.83
H(1)=5.683; *p*=.017*					
Work experience	0-5 *vs* 6-10 (0,1)	4056.00	-1.79	.073	98.61 *vs* 111.85
	0-5 vs 11-15 (0,2)	1053.50	-1.178	.239	76.69 vs 87.97
	0-5 vs 16-20 (0,3)	1122.00	-.727	.467	77.19 vs 84.17
	0-5 vs 21-25 (0,4)	1654.00	-1.351	.177	81.07 vs 92.43
	0-5 vs 26-30 (0,5)	1438.00	-1.838	.066	79.50 vs 95.19
	0-5 vs 31-35 (0,6)	1132.50	-1.473	.141	77.27 vs 90.88
	0-5 vs 36-40 (0,7)	118.50	-1.467	.142	69.86 vs 99.50
	0-5 vs > 41 (0,8)	126.50	-.217	.828	70.08 vs 64.75
	6-10 vs 11-15 (1,2)	602.00	-.135	.893	43.35 vs 44.06
	6-10 vs 16-20 (1,3)	588.00	-.320	.749	43.85 vs 42.17
	6-10 vs 21-25 (1,4)	944.00	-.082	.935	48.38 vs 48.79
	6-10 vs 26-30 (1,5)	828.00	-.611	.541	46.68 vs 49.65
	6-10 vs 31-35 (1,6)	650.00	-.382	.702	44.06 vs 46.00
	6-10 vs 36-40 (1,7)	72.00	-1.100	.271	35.56 vs 46.00
	6-10 vs >41 (1,8)	54.00	-.622	.534	35.71 vs 28.50
	11-15 vs 16-20 (2,3)	153.00	-.357	.721	19.00 vs 18.00
	11-15 vs 21-25 (2,4)	250.00	-.058	.954	23.61 vs 23.43
	11-15 vs 26-30 (2,5)	223.00	-.350	.726	21.89 vs 22.92
	11-15 vs 31-35 (2,6)	175.00	-.192	.848	19.22 vs 19.75
	11-15 vs 36-40 (2,7)	19.50	-1.021	.307	10.58 vs 13.50
	11-15 vs > 41 (2,8)	14.00	-.634	.526	10.72 vs 8.50
	16-20 vs 21-25 (3,4)	240.00	-.339	.735	22.83 vs 23.93
	16-20 vs 26-30 (3,5)	210.00	-.742	.458	21.17 vs 23.42

		16-20 vs 31-35 (3,6)	165.00	-.558	.577	18.67 vs 20.25
		16-20 vs 36-40 (3,7)	18.00	-1.155	.248	10.50 vs 14.00
		16-20 vs >41 (3,8)	15.00	-.457	.648	10.67 vs 9.00
		21-25 vs 26-30 (4,5)	344.00	-.456	.648	26.79 vs 28.27
		21-25 vs 31-35 (4,6)	270.00	-272	.786	24.14 vs 25.00
		21-25 vs 36-40 (4,7)	30.00	-1.057	.290	15.57 vs 20.00
		21-25 vs > 41 (4,8)	22.00	-.628	.530	15.71 vs 12.50
		26-30 vs 31-35 (5,6)	255.00	-.150	.881	23.69 vs 23.25
		26-30 vs 36-40 (5,7)	30.00	-.918	.359	14.65 vs 18.00
		26-30 vs > 41 (5,8)	19.00	-.832	.405	14.77 vs 11.00
		31-35 vs 36-40 (6,7)	22.50	-.957	.338	11.63 vs 14.50
		31-35 vs > 41 (6,8)	15.00	-.74.	.460	11.75 vs 9.00
		36-40 vs > 41 (7,8)	1.50	-1.225	.221	3.50 vs 2.25
	Professional training					
		<1 vs 1-5 h/week (0-1)	9806.00	-1.414	.157	142.40 *vs* 154.22
		<1 vs 6-10 h/week (0-2)	1146.50	-.310	.756	69.63 *vs* 72.18
		<1 vs >11 h/week (0-3)	105.50	-.325	.745	61.11 *vs* 54.25
		1-5 vs 6-10 h/week (1-2)	100.43	-.390	.696	100.43 *vs* 96.18
		1-5 vs >11 h/week (1-3)	144.50	-.585	.595	91.19 *vs* 73.75
		6-10 vs >11 h/week (2-3)	17.00	-.411	.681	11.65 *vs* 10.00
	Geographical area					
		North vs Center (0,1)	5903.50	-1.263	.207	118.81 *vs* 109.79
		North vs South (0,2)	46.21	-.938	.348	102.90 *vs* 96.69
		Center vs South (1,2)	5530.50	-.256	.798	106.71 *vs* 108.53

U: U di Mann-Whitney; H: (Degrees of freedom); * statistically significant value
Behav_1: collaboration with the pharmacist

Table S19: Results of the Kruskal-Wallis test and post-hoc comparisons performed using the Mann-Whitney test: behaviour section

Variable		*Variable and categories*	*U*	*Z*	*p*	*Average Rank*
Behav_2	H(1)=6.281; *p*= < .012*					
	Age	20-25 *vs* 26-30 (0-1)	1952.50	-.089	.929	64.72 *vs* 64.36
		20-25 vs 31-35 (0-2)	1381.50	-.814	.416	53.09 vs 55.76
		20-25 vs 36-40 (0-3)	421.50	-.924	.355	34.26 vs 37.08
		20-25 vs 41-45 (0,4)	616.00	-2.040	.041*	38.08 vs 43.50
		20-25 vs 46-50 (0,5)	883.50	-1.833	.067	43.32 vs 48.35
		20-25 vs 51-55 (0,6)	799.00	-1.145	.252	41.67 vs 45.00

	20-25 vs 56-60 (0,7)	286.00	-1.404	.160	31.61 vs 36.00
	20-25 vs >61 (0,8)	66.00	-.681	.496	27.29 vs 31.00
	26-30 vs 31-35 (1,2)	2073.50	-.970	.332	65.93 vs 69.62
	26-30 vs 36-40 (1,3)	632.50	-.998	.318	47.21 vs 51.36
	26-30 vs 41-45(1,4)	924.00	-2.104	.035*	51.00 vs 58.50
	26-30 vs 46-50 (1,5)	1325.50	-1.950	.051	56.21 vs 63.01
	26-30 vs 51-55 (1,6)	1199.00	-1.263	.206	54.57 vs 59.24
	26-30 vs 56-60 (1,7)	429.00	-1.446	.148	44.57 vs 51.00
	26-30 vs >61 (1,8)	99.00	-.700	.484	40.29 vs 46.00
	31-35 vs 36-40 (2,3)	496.50	-.436	.663	37.71 vs 38.92
	31-35 vs 41-45 (2,4)	728.00	-1.606	.108	41.77 vs 45.50
	31-35 vs 46-50 (2,5)	1042.50	-1.228	.220	47.29 vs 50.27
	31-35 vs 51-55 (2,6)	941.00	-.498	.619	45.51 vs 46.82
	31-35 vs 56-60 (2,7)	338.00	-1.100	.271	34.93 vs 38.00
	31-35 vs >61 (2,8)	78.00	-.531	.595	30.37 vs 33.00
	36-40 vs 41-45 (3,4)	238.00	-1.247	.212	22.72 vs 24.00
	36-40 vs 46-50 (3,5)	340.50	-.566	.572	28.42 vs 29.27
	36-40 vs 51-55 (3,6)	284.00	-.580	.562	26.56 vs 26.47
	36-40 vs 56-60 (3,7)	110.50	-.850	.395	15.64 vs 16.50
	36-40 vs >61 (3,8)	25.50	-.408	.683	10.92 vs 11.50
	41-45 vs 46-50 (4,5)	532.00	-.847	.397	34.50 vs 33.64
	41-45 vs 51-55 (4,6)	404.00	-1.318	.188	32.50 vs 30.68
	41-45 vs 56-60 (4,7)	182.00	.000	1.00	21.00 vs 21.00
	41-45 vs >61 (4,8)	42.00	.000	1.00	16.00 vs 16.00
	46-50 vs 51-55 (5,6)	641.00	-.708	.479	37.56 vs 36.35
	46-50 vs 56-60 (5,7)	247.00	-.577	.564	26.33 vs 27.00
	46-50 vs >61 (5,8)	57.00	-.277	.782	21.46 vs 22.00
	51-55 vs 56-60 (6,7)	208.00	-.884	.377	23.62 vs 25.00
	51-55 vs >61 (6,8)	48.00	-.426	.670	18.91 vs 20.00
	56-60 vs >61 (7,8)	19.50	.000	1.00	8.50 vs 8.50
H(1)=6.757; p=.009*					
Work experience	0-5 vs 6-10 (0,1)	4525.00	-.620	.535	102.03 vs 104.96
	0-5 vs 11-15 (0,2)	1157.50	-.779	.436	77.45 vs 82.19
	0-5 vs 16-20 (0,3)	1157.50	-.779	.436	77.45 vs 82.19
	0-5 vs 21-25 (0,4)	1762.50	-1.282	.200	81.86 vs 88.55

	0-5 vs 26-30 (0,5)	1710.00	-.593	.553	81.48 vs 84.73
	0-5 vs 31-35 (0,6)	1210.00	-1.608	.108	77.83 vs 87.00
	0-5 vs 36-40 (0,7)	181.50	-.627	.531	70.32 vs 78.50
	0-5 vs > 41 (0,8)	121.00	-.512	.609	69.88 vs 78.00
	6-10 vs 11-15 (1,2)	592.00	-.448	.654	43.21 vs 44.61
	6-10 vs 16-20 (1,3)	592.00	-.448	.654	43.21 vs 44.61
	6-10 vs 21-25 (1,4)	902.00	-.895	.371	47.76 vs 50.29
	6-10 vs 26-30 (1,5)	874.00	-.175	.861	47.35 vs 47.88
	6-10 vs 31-35 (1,6)	620.00	-1.368	.171	43.62 vs 47.50
	6-10 vs 36-40 (1,7)	93.00	-.534	.593	35.87 vs 39.00
	6-10 vs >41 (1,8)	62.00	-.436	.663	35.41 vs 38.50
	11-15 vs 16-20 (2,3)	162.00	.000	1.00	18.50 vs 18.50
	11-15 vs 21-25 (2,4)	247.00	-.319	.750	23.22 vs 23.68
	11-15 vs 26-30 (2,5)	229.00	-.273	.785	22.78 vs 22.31
	11-15 vs 31-35 (2,6)	170.00	-1.054	.292	18.94 vs 20.00
	11-15 vs 36-40 (2,7)	25.50	-.408	.683	10.92 vs 11.50
	11-15 vs > 41 (2,8)	17.00	-.333	.739	10.44 vs 11.00
	16-20 vs 21-25 (3,4)	247.00	-.319	.750	23.22 vs 23.68
	16-20 vs 26-30 (3,5)	229.00	-.273	.785	22.78 vs 22.31
	16-20 vs 31-35 (3,6)	170.00	-1.054	.292	18.94 vs 20.00
	16-20 vs 36-40 (3,7)	25.50	-.408	.683	10.92 vs 11.50
	16-20 vs >41 (3,8)	17.00	-.333	.739	10.44 vs 11.00
	21-25 vs 26-30 (4,5)	349.00	-.654	.513	28.04 vs 26.92
	21-25 vs 31-35 (4,6)	270.00	-.845	.398	24.14 vs 25.00
	21-25 vs 36-40 (4,7)	40.50	-.327	.743	15.95 vs 16.50
	21-25 vs > 41 (4,8)	27.00	-.267	.789	15.46 vs 16.00
	26-30 vs 31-35 (5,6)	240.00	-1.254	.210	22.73 vs 24.50
	26-30 vs 36-40 (5,7)	36.00	-.489	.625	14.88 vs 16.00
	26-30 vs > 41 (5,8)	24.00	-.400	.689	14.42 vs 15.50
	31-35 vs 36-40 (6,7)	30.00	.000	1.00	12.00 vs 12.00
	31-35 vs > 41 (6,8)	20.00	.000	1.00	11.50 vs 11.50
	36-40 vs > 41 (7,8)	3.00	.000	1.00	3.00 vs 3.00
	Professional training				
	<1 vs 1-5 h/week (0-1)	10052.0	-1.711	.087	144.47 vs 152.84
	<1 vs 6-10 h/week (0-2)	1169.00	-.288	.820	69.82 *vs* 71.05
	<1 vs >11 h/week (0-3)	105.00	-.514	..607	60.88 *vs* 68.00

	1-5 vs 6-10 h/week (1-2)	1721.00	-.660	.509	100.39 *vs* 96.55
	1-5 vs >11 h/week (1-3)	168.00	-.361	.718	90.94 *vs* 96.50
	6-10 vs >11 h/week (2-3)	18.00	-.458	.647	11.40 *vs* 12.50
Geographical area					
	North vs Center (0,1)	6299.00	-.537	.591	115.08 *vs* 113.06
	North vs South (0,2)	4572.00	-1.773	.076	103.37 *vs* 96.16
	Center vs South (1,2)	5319.00	-1.329	.184	110.04 *vs* 104.19

U: U di Mann-Whitney; H: (Degrees of freedom); * statistically significant value
Behav_2: health documentation

Table S20: Results of the Kruskal-Wallis test and post-hoc comparisons performed using the Mann-Whitney test: behaviour section

Variable		*Variable and categories*	*U*	*Z*	*p*	*Average Rank*
Behav_3	Age<br>H(1)=.466; *p*=.495					
	Work experience<br>H(1)= 1.153; *p*= .283					
	Professional training	<1 *vs* 1-5 h/week (0-1)	10169.50	-1.095	.273	145.46 *vs* 152.19
		<1 vs 6-10 h/week (0-2)	1092.50	-.880	.379	70.82 vs 65.13
		<1 vs >11 h/week (0-3)	79.50	-1.224	.221	61.33 vs 41.25
		1-5 vs 6-10 h/week (1-2)	1562.50	-1.570	.116	101.27 vs 88.63
		1-5 vs >11 h/week (1-3)	111.50	-1.588	.112	91.38 vs 57.25
		6-10 vs >10 (2-3)	15.00	-.740	.460	11.75 vs 9.00
	Geographical area					
		North vs Center (0,1)	5700.50	-2.256	.024*	120.72 vs 108.11
		North vs South (0,2)	4857.50	-.325	.745	100.67 vs 99.23
		Center vs South (1,2)	5083.00	-1.848	.065	103.01 vs 113.34

U: U di Mann-Whitney; H: (Degrees of freedom); * statistically significant value
Behav_3: support guidelines

Table S21: Results of the Kruskal-Wallis test and post-hoc comparisons performed using the Mann-Whitney test: behaviour section

Variable		*Variable and categories*	*U*	*Z*	*p*	*Average Rank*
Behav_4	Age<br>H(1)=2.232; *p*=.135					
	Work experience<br>H(1)= 2.689; *p*= .101					
	Professional training	<1 *vs* 1-5 h/week (0-1)	9603.00	-2.31	.020*	140.70 *vs* 155.35
		<1 vs 6-10 h/week (0-2)	1059.00	-1.17	.251	68.90 vs 76.55

		<1 vs >11 h/week (0-3)	94.00	-.725	.469	60.79 vs 73.50
		1-5 vs 6-10 h/week (1-2)	1769.00	-.158	.874	99.88 vs 101.05
		1-5 vs >11 h/week (1-3)	159.00	-.500	.617	90.89 vs 101.00
		6-10 vs >10 (2-3)	18.00	-.458	.647	11.40 vs 12.50
	Geographical area					
		North vs Center (0,1)	6194.50	-.777	.437	111.94 vs 115.81
		North vs South (0,2)	4619.50	-1.171	.242	102.92 vs 96.67
		Center vs South (1,2)	5081.50	-1.965	.049*	112.00 vs 101.64

U: U di Mann-Whitney; H: (Degrees of freedom); * statistically significant value
Behav_4: medication reconciliation

Table S22: Results of the Kruskal-Wallis test and post-hoc comparisons performed using the Mann-Whitney test: behaviour section

Variable		*Variable and categories*	*U*	*Z*	*p*	*Average Rank*
Behav_5	Age<br>H(1)=.846; *p*=.358					
	Work experience<br>H(1)= 1.228; *p*= .268					
	Professional training	<1 *vs* 1-5 h/week (0-1)	10259.50	-.951	.342	146.21 *vs* 151.68
		<1 vs 6-10 h/week (0-2)	1079.50	-1.140	.254	69.07 vs 75.53
		<1 vs >11 h/week (0-3)	76.50	-1.402	.161	61.36 vs 39.75
		1-5 vs 6-10 h/week (1-2)	1689.50	-.790	.430	99.44 vs 105.03
		1-5 vs >11 h/week (1-3)	108.50	-1.762	.078	91.39 vs 55.75
		6-10 vs >10 (2-3)	11.00	-2.062	.039*	11.95 vs 7.00
	Geographical area					
		North vs Center (0,1)	5859.50	-1.814	.070	119.22 vs 109.43
		North vs South (0,2)	4629.50	-1.616	.106	97.17 vs 103.22
		Center vs South (1,2)	4799.00	-3.204	.001*	100.66 vs 116.40

U: U di Mann-Whitney; H: (Degrees of freedom); * statistically significant value
Behav_5: use of hydroalcoholic gel

Table S23: Results of the Kruskal-Wallis test and post-hoc comparisons performed using the Mann-Whitney test: behaviour section

Variable		*Variable and categories*	*U*	*Z*	*p*	*Average Rank*
Behav_6	Age H(3)=2.530; *p*= .112					
	H(3)=3.915; *p*=.048*					
	Work experience	0-5 *vs* 6-10 (0,1)	4620.50	-.182	.855	103.27 *vs* 102.45
		0-5 vs 11-15 (0,2)	1213.00	-.218	.827	78.15 vs 76.89
		0-5 vs 16-20 (0,3)	1184.50	-.546	.585	77.65 vs 80.69
		0-5 vs 21-25 (0,4)	1826.00	-.758	.448	83.67 vs 79.71

	0-5 vs 26-30 (0,5)	1607.50	-1.449	.147	83.27 vs 75.33
	0-5 vs 31-35 (0,6)	952.00	-3.733	.001*	82.05 vs 58.10
	0-5 vs 36-40 (0,7)	186.00	-.558	.577	70.36 vs 77.00
	0-5 vs > 41 (0,8)	124.00	-.456	.648	69.91 vs 76.50
	6-10 vs 11-15 (1,2)	607.00	-.100	.920	43.57 vs 43.22
	6-10 vs 16-20 (1,3)	583.00	-.612	.541	43.07 vs 45.11
	6-10 vs 21-25 (1,4)	914.00	-.555	.579	49.06 vs 47.14
	6-10 vs 26-30 (1,5)	805.00	-1.155	.248	48.66 vs 44.46
	6-10 vs 31-35 (1,6)	478.00	-3.088	.002*	47.47 vs 34.40
	6-10 vs 36-40 (1,7)	91.50	-.581	.561	35.85 vs 39.50
	6-10 vs >41 (1,8)	61.00	-.475	.635	35.40 vs 39.00
	11-15 vs 16-20 (2,3)	153.00	-.595	.552	18.00 vs 19.00
	11-15 vs 21-25 (2,4)	244.00	-.309	.758	23.94 vs 23.21
	11-15 vs 26-30 (2,5)	215.00	-.716	.474	23.56 vs 21.77
	11-15 vs 31-35 (2,6)	128.00	-1.993	.046*	22.39 vs 16.90
	11-15 vs 36-40 (2,7)	24.00	-.592	.554	10.83 vs 12.00
	11-15 vs > 41 (2,8)	16.00	-.484	.628	10.39 vs 11.50
	16-20 vs 21-25 (3,4)	230.00	-.918	.358	24.72 vs 22.71
	16-20 vs 26-30 (3,5)	202.00	-1.285	.199	24.28 vs 21.27
	16-20 vs 31-35 (3,6)	118.00	-2.461	.014*	22.94 vs 16.40
	16-20 vs 36-40 (3,7)	25.50	-.408	.683	10.92 vs 11.50
	16-20 vs >41 (3,8)	17.00	-.333	.739	10.44 vs 11.00
	21-25 vs 26-30 (4,5)	346.00	-.483	.629	28.14 vs 26.81
	21-25 vs 31-35 (4,6)	208.00	-2.007	.045*	27.07 vs 20.90
	21-25 vs 36-40 (4,7)	36.00	-.69.	.490	15.79 vs 18.00
	21-25 vs > 41 (4,8)	24.00	-.565	.572	15.36 vs 17.50
	26-30 vs 31-35 (5,6)	206.00	-1.534	.125	25.58 vs 20.80
	26-30 vs 36-40 (5,7)	31.50	-.820	.412	14.71 vs 17.50
	26-30 vs > 41 (5,8)	21.00	-.672	.502	14.31 vs 17.00
	31-35 vs 36-40 (6,7)	18.00	-1.327	.185	11.40 vs 16.00
	31-35 vs > 41 (6,8)	12.00	-1.095	.273	11.50 vs 11.50
	36-40 vs > 41 (7,8)	3.00	.000	1.00	3.00 vs 3.00
Professional training					
	<1 *vs* 1-5 h/week (0-1)	10319.00	-.797	.426	146.71 *vs* 151.35
	<1 vs 6-10 h/week (0-2)	1181.50	-.084	.933	70.07 vs 69.58
	<1 vs >11 h/week (0-3)	102.00	-.574	.566	60.86 vs 69.50

	1-5 vs 6-10 h/week (1-2)	1721.50	-.506	.613	100.38 vs 96.58
	1-5 vs >11 h/week (1-3)	159.00	-.500	.617	90.89 vs 101.00
	6-10 vs >10 (2-3)	17.00	-.576	.565	11.35 vs 13.00
Geographical area					
	North vs Center (0,1)	5657.50	-2.447	.014*	106.87 vs 120.24
	North vs South (0,2)	4124.50	-3.295	.001*	92.41 vs 108.65
	Center vs South (1,2)	5371.00	-1.215	.224	105.39 vs 110.25

U: U di Mann-Whitney; H: (Degrees of freedom); * statistically significant value
Behav_6: vital signs
